# Datastorr: a workflow and package for delivering successive versions of 'evolving data' directly into R

**DOI:** 10.1093/gigascience/giz035

**Published:** 2019-05-01

**Authors:** Daniel S Falster, Richard G FitzJohn, Matthew W Pennell, William K Cornwell

**Affiliations:** 1 Evolution & Ecology Research Centre, and School of Biological, Earth and Environmental Sciences, University of New South Wales, Sydney NSW 2052, Australia; 2 Department of Infectious Disease Epidemiology, Imperial College London, Faculty of Medicine, Norfolk Place, London W2 1PG, UK; 3 Department of Zoology and Biodiversity Research Centre, University of British Columbia, Vancouver, BC V6T 1Z4, Canada

**Keywords:** data sharing, version control, semantic versioning

## Abstract

The sharing and re-use of data has become a cornerstone of modern science. Multiple platforms now allow easy publication of datasets. So far, however, platforms for data sharing offer limited functions for distributing and interacting with evolving datasets— those that continue to grow with time as more records are added, errors fixed, and new data structures are created. In this article, we describe a workflow for maintaining and distributing successive versions of an evolving dataset, allowing users to retrieve and load different versions directly into the R platform. Our workflow utilizes tools and platforms used for development and distribution of successive versions of an open source software program, including version control, GitHub, and semantic versioning, and applies these to the analogous process of developing successive versions of an open source dataset. Moreover, we argue that this model allows for individual research groups to achieve a dynamic and versioned model of data delivery at no cost.

## Introduction

Sharing of a high-quality dataset—a collection of measurements, stored in 1 or several files—is now considered a first-class scientific output. Increasingly, funding bodies, publishers, and scientific social norms are recognizing the value of sharing datasets, including as stand-alone products without any accompanying analyses [1–5]. Evidence of this trend is seen in the increasing numbers of stand-alone “data papers” appearing in both standard domain-level journals and specialized data journals. Yet, while the last decade has witnessed a rapid and exciting change in attitudes towards data sharing, the scientific community is still grappling with how to effectively maintain and distribute open source datasets [1, 4, 6, 7, 8, 9, 10, 11, 12]. In particular, in some areas, such as our own area of ecology and evolution, we are only starting to support the fact that some high-quality datasets may be evolving entities [12].

An evolving (or "living") dataset is one that is subject to occasional or recurrent change. Typical changes may include improving the quality of existing data, adding new data, re-structuring the dataset content, or integrating with other datasets. For example, a dataset on biological organisms might be expanded through the addition of new records or improved through the correction of spelling mistakes in taxonomic names. In some cases, datasets may be expected to continue to evolve over extended periods (e.g., [13]. Evolving datasets are never “finished,” and as such there is no “master” or “canonical” version. Rather, as research around a data product grows, there might be many valid versions produced. Even datasets that are not initially envisioned as evolving may become so as minor errors are identified and corrected during use. In either case, the most recent version of the dataset will typically contain the best available information, but there are still reasons to go back to previous versions: to replicate previous analyses or to work on a stable version for downstream analyses or visualization.

A common approach taken by those maintaining an evolving dataset is to release sequential versions of the dataset, each containing a snapshot of the dataset at the time of release (e.g., [12, 14, 15, 16]. Ideally, the latest versions of an evolving dataset would be immediately available to all users across the globe, along with notes describing the changes when compared to previous versions. For the sake of reproducibility, previous versions of the dataset should be archived and remain available. In the recent past, small research groups have solved the issue of versioning data internally and informally, e.g., by e-mailing around the latest version. However, as science grows and moves towards more systematic sharing of data, scalable solutions are needed to distribute dataset versions to a wider variety of users.

One approach taken by large research consortia has been to create dedicated web servers for archiving and delivering of data. Projects such as the Sloan Digital Sky Survey [17] have sophisticated infrastructure and processes for distributing successive versions of very large datasets [16]. The issue of updating data has also been addressed in some centralized repositories, like genetic sequences (via GenBank), where new data can be added and there exist abilities to correct errors in existing records. Yet these web databases require a level of funding and technological infrastructure that is beyond most research groups.

Almost all research projects are smaller, and these currently rely on more generic data repositories for distributing data. A common approach for distributing a dataset is to release it under a Digital Object Identifier (DOI) in a stand-alone data repository, such as DataDryad, Figshare, and Zenodo. While these platforms did not all initially support versioning of datasets, they now support multiple versions of a dataset, either under a single or different DOIs [18]. Yet, while these new features in principle allow users to access multiple versions of a dataset, the release, discovery, and access to multiple versions of a dataset is not always straightforward.

We believe that more can be done to streamline the distribution of a potentially large number of dataset versions to users, especially for small research teams with limited budgets. There are at least 3 challenges. First, dataset developers need a cheap—ideally free—and reliable system to create and distribute versions of an evolving dataset with low technical overhead. Second, users need an easy mechanism to discover the existence of new (or all) versions of an evolving dataset. Third, users need a mechanism to retrieve specific versions. For those using a computational language such as R [19], all versions of an evolving dataset would ideally be both accessible and discoverable directly from within R.

In this article we outline how emerging technologies from software development (Table 1) can be used to address these challenges, enabling small research groups to create and maintain a stream of versions for small-to-medium sized datasets (≤2 Gb), and distribute these directly into the R computational environment for a potentially unlimited number of users at zero financial cost and minimal technical overhead. To achieve this we developed a new R package called datastorr, which together with other technologies allows for easy and scalable delivery of successive versions of an evolving dataset directly into R. At the time of publishing this article, this workflow was being used to distribute versions of datasets across a wide range of topics (Table 2), suggesting a potentially wide domain of application.

**Table 1.** Overview of technologies used to maintain, store, and distribute versions of an evolving dataset as described in this article

**Technology**	**Description**
git	Open source version control system used for tracking progressive changes in a set of text files, typically computer code but also data
GitHub	A commercial web platform [21] for sharing, visualizing, and managing git repositories. Includes ability to browse the "history," "issue" tracking, and ability to host "releases." Also has a well-developed API enabling programmatic access to dataset releases
R	Widely used and open source language for data processing and statistical analysis[19]
datastorr	A package in R used to fetch releases of an evolving dataset hosted on GitHub[22]
Semantic versioning	The process of assigning unique version numbers in a particular format to successive versions of a digital product[23]; traditionally applied to software but here to an evolving dataset

API: application programming interface.

**Table 2.** Example datasets currently delivered using the datastorr package for R

**GitHub repository**	**Dataset description**
traitecoevo/taxonlookup [24]	Taxonomy of world’s land plants [15]
traitecoevo/growthform [25]	Growth form of world’s land plants [26]
traitecoevo/baad.data [27]	Size dimensions of plants for many species from across the world [14]
ecohealthalliance/cites [28]	Trade details from Convention on International Trade in Endangered Species (CITES)
madams1/nbadata [29]	Statistics from the National Basketball Association (NBA) seasons 1996-97 to 2016-17
madams1/floridainmates [30]	Statistics on Florida state’s inmate population, from Florida Department of Corrections
traitecoevo/fungaltraits [31]	Traits of world’s fungi species [32]

## A Lightweight, Cheap, and Scalable Workflow for Delivering Versions of an Evolving Dataset into R

**Figure 1:**
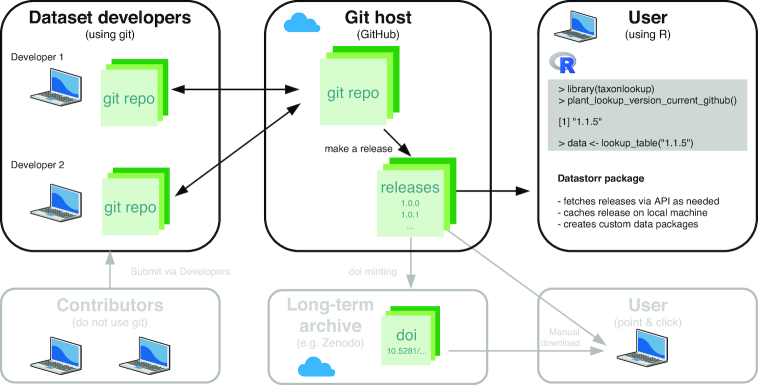
Overview of the workflow, different parties, and technologies involved in maintaining and distributing versions of an evolving dataset via datastorr. Core features of our approach are shown with black boxes and arrows. Optional extensions are shown in grey (see Discussion for details).

In brief, the workflow we present here borrows best practices for software development [20] and applies them to the challenge of maintaining and distributing versions of an evolving dataset. Our approach envisions multiple parties involved in the creation and/or use of a versioned dataset, including developers, contributors, and users (Fig. 1). Each of these will likely have different goals and requirements (see Table 3). When building a piece of software, developers maintain a core set of code that produces the binary executable file that is eventually installed on a user’s local computer. Analogously, developers of an evolving dataset maintain a core set of files (the “code”), which produces an organized dataset that can be “installed” (i.e., loaded) on a user’s local computer. In the development of either software or data, successive versions—called “releases”—are distributed as snapshots of the generated product at a particular point in time.

**Table 3.** Goals and requirements of different parties involved in creating and using an evolving dataset

**Group**	**Primary goal**	**Requirements**
Developers	Create and distribute versions of an evolving dataset	Low technical overhead, low initial and ongoing cost and maintenance, easy workflow for releasing new versions, enable user feedback in error checking and contributions, long-term preservation
Contributors	Contribute to future versions of an evolving dataset	Add new data, report errors in existing data
Users (all)	Gain easy access to all versions of an evolving dataset	Access metadata and background information, access to all versions of a dataset, ability to provide feedback and contribute, long-term stability
Users (programmatic)	As above via machine access	Programmatic access to all versions of an evolving dataset, reproduce products using specific versions of an evolving dataset, easy installation

The similarity in workflow between software and data allows us to re-purpose some of the same technological platforms that are used to maintain and distribute versions of a software product to maintain and distribute versions of an evolving dataset (Table 1). Importantly, these tools are available free of charge for open source projects and already well developed—ensuring high-level performance and stability. Moreover, the combination of technologies allows us to address the goals and requirements of the different parties involved in the creation and use of a versioned dataset (see Table 3).

An overview of the proposed system is as follows.

- Raw data files are stored under version control in a git repository—a free and leading version control system used in software development—by the dataset developers. All the files that go together to build a single dataset are stored in the repository, together with any code used to manipulate these files to create the dataset that is ultimately distributed.
- Changes to the raw data files and code are tracked by the developers using git’s ability to make “commits”—granular and annotated snapshots of the source files over time.
- The git repository is hosted on GitHub, a leading platform for hosting, enabling multiple developers or other contributors to work collaboratively on improving a dataset (Fig. 1).
- Developers use the files in the repository to make a release of the dataset—a snapshot of the generated data product at a particular commit—and upload these to GitHub, where they are hosted alongside the raw files and (optionally) labelled using “semantic versioning.” The version labels indicate both the ordering of versions and the magnitude of change expected between different versions.
- Using the datastorr package, users can both retrieve a list of all available versions of the dataset, and retrieve particular versions of the dataset on demand, and load them directly into R.
- Those not using R can also access versions from GitHub.

Below we elaborate on each of the different technologies.

### Version control

Version control, primarily an open source variety called git, has become widespread in software development. In practice, version control tracks line-by-line changes in text files and creates and maintains a history of those changes. Increasingly version control has been applied to scientific code and also data management, especially for small-to-medium sized datasets [8, 9, 33]. git is attractive for data management because it tracks all changes in monitored files, provided these are saved in text format (e.g., “.csv”, “.tsv”, “.txt”; with some tricks git can also indicate changes in some other file types such as “.xlsx”). It allows users to annotate commits with informative messages detailing the rationale for those changes. The “history” of commits is also visible to anyone interacting with the repository. In its present form, git can handle individual data files at least up to 100 MB, which includes a large fraction of scientific cases.

As a general strategy for tracking a dataset under version control with git, we recommend the following:

- Developers establish a separate git repository for each dataset to be distributed.
- Saving data in their rawest form. Some datasets might only have a single file. Others may have many files that get manipulated or combined in some way to produce a unified product.
- Where possible, saving all files as plain text, so that git can identify line-by-line changes. For example, one should save tabular data as a “.csv”. While this approach works well for small-to-intermediate sized files, those with larger files may prefer to use a compressed format to reduce repository size and bandwidth.
- Including in the git repository any code needed to manipulate or compile the raw data files into the final dataset. For example, one might combine many independent datasets into 1 unified dataset.
- Documenting any changes in the dataset by making a commit in the git repository, with an informative message outlining why the change was made.

### Hosting and distributing versions of an evolving dataset

Datasets stored under version control via git reach their real potential when hosted at a suitable internet hosting service [9, 33]. Here we focus on the platform GitHub (Table 1). Hosting of a git repository enables dataset developers to connect with other potential contributors and also users (Fig. 1). These platforms are designed to work with git repositories and thus offer many helpful features, such as the ability to record issues, host documentation, or review edits over time.

Another notable feature of GitHub is the ability to host a stream of releases from the dataset, alongside the git repository containing all the raw files. Each release is linked to a specific commit in the git repository history and occurs at points where the dataset developer decided to generate a new version of the data for distribution. While users could in principle download the entire git repository, most of the time, what they want are the releases.

Deciding when to make a new release is at the discretion of the dataset developer. In practice, one makes fewer releases than one does commits into the git repository, although there is nothing stopping developers from releasing a new version for every commit. The flexibility here allows developers to do internal work between releases and only release the data to users when the revision represents a clear improvement on the previous release.

Another important consideration is that websites like GitHub naturally cater to 2 types of data users accessing the data: those who interact with the data via point-and-click downloading and those who use programmatic interaction (Fig. 1, Table 3). Specifically, GitHub releases can be downloaded directly by users or accessed programmatically via the GitHub API.

### Semantic versioning

To realize the full benefits of a versioned controlled dataset, users should be able to easily intuit the types of changes that have occurred among versions. Because software development has effectively already dealt with a similar problem in the labelling of software releases, we suggest adopting the best practices from that field.

**Figure 2:**
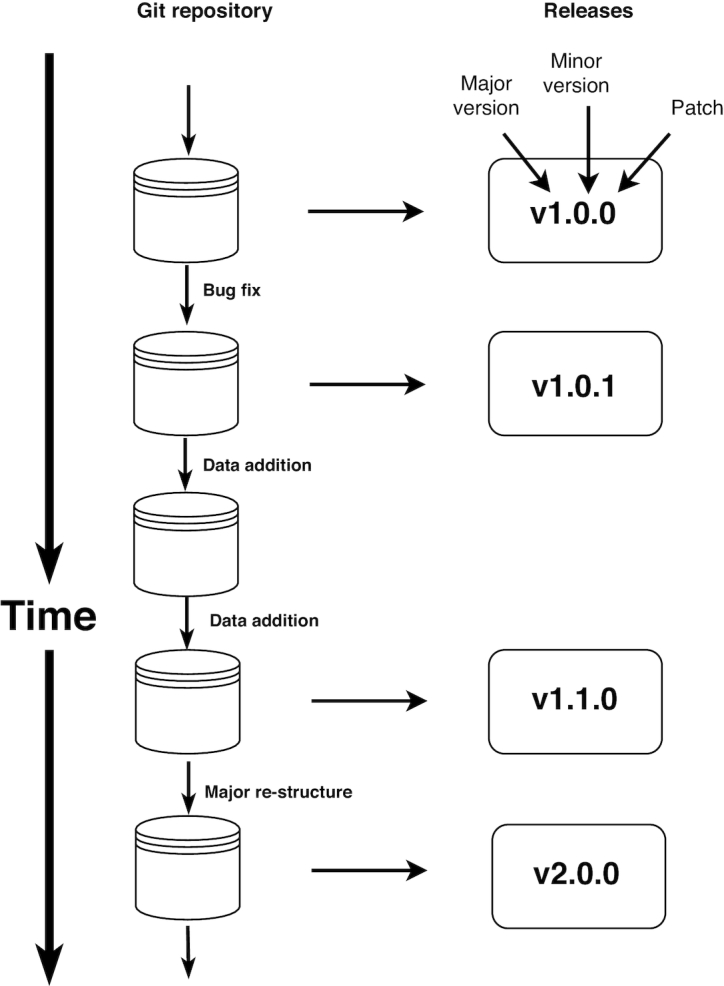
Semantic versioning allows dataset developers to communicate to users the types of changes that have occurred between successive versions of an evolving dataset, using a tri-digit label where increments in a number indicate major, minor, and patch-level changes, respectively. See text for further details.

Specifically, we suggest adapting the process of semantic versioning, developed for labelling successive releases of software (see semver.org [23]), to labelling of successive releases of an evolving dataset (Fig 2). In semantic versioning of software, a tri-digit label of the form “X.Y.Z” is applied to each version, where X, Y, and Z are non-negative integers, e.g., version “2.1.2”. Although everyday practice may differ, the guidelines at semver.org [23] suggest labels are incremented in a particular way, determined by changes in the public API for the software.

Although the analogy to software is not perfect, datasets can also be thought of as having an “interface,” determined by the structure of the dataset, which dictates how users interact with the resource. For example, in tabular data the structure is determined by the names of different files, the column labels within each, and the presence of different subgroups within the table (as indicated by labels in particular columns). Successive versions of a dataset can then be labelled in a manner analogous to that of software, determined by the structure of the dataset and changes in that structure.

There are 2 natural advantages of adapting the process of semantic versioning for dataset development. The first is that it enables natural ordering of releases. The second is that it enables developers to signal the type and magnitude of change that occurred in the product between successive versions. Seeing a series of version numbers, users of an evolving dataset know the developer’s view on the type and/or magnitude of change between versions.

Drawing inspiration from the guidelines for semantic versioning of software at semver.org [23], we suggest the following guidelines for labelling of a dataset with semantic versioning:

- Clearly communicate the structure of the dataset in the metadata or landing page. This includes file types, data type, and element names.
- Use versions beginning with “0.Y.Z” to indicate products where the structure is still in development.
- Version “1.0.0” defines the structure.
- Once defined, increment version numbers to communicate any changes to the structure.
- Increment the ”major” version when you make changes to the structure that are likely incompatible with any code written to work with previous versions. Such changes may include revising the file names, the structure of the dataset, or changing element names (e.g., column headers). Substantial additions of data might also be considered a major change to structure, especially where they add new subgroups to the dataset.
- Increment the ”minor” version to communicate any changes to the structure that are likely to be compatible with any code written to work with the previous versions (i.e., allows code to run without error). Such changes might involve adding new data within the existing structure, so that the previous dataset version exists as a subset of the new version. For tabular data, this includes adding columns or rows. On the other hand, removing data should constitute a major version because records previously relied on may no longer exist.
- Increment the ”patch” version to communicate correction of errors in the actual data, without any changes to the structure. Such changes are unlikely to break or change analyses written with the previous version in a substantial way.
- Once a dataset version has been released, do not modify it. Further modifications are released under a new version number.

While it is hoped that the aforementioned guidelines help users in understanding the types of changes that have occurred between successive versions of a dataset, any change in a dataset may alter the results of a user's analysis in non-trivial ways. Unlike developers of software, developers of a dataset cannot guarantee full backwards compatibility, i.e., that certain results will remain unchanged in updated versions. We suggest that responsibility for verifying how different versions of an evolving dataset influence their particular analysis or use thus always remains with the user, even if the change is as small as the simple application of a so-called “patch.” While further work—and likely experience—is needed to refine the process of semantic versioning for datasets to further develop understanding between data developers and data users of what different changes imply, semantic versioning still provides a more nuanced way to communicate from the developer to the user on the types of change they could expect.

### Loading data versions directly into R using the datastorr package

For efficient usage and to aid reproducibility, many users will want access to all versions of any particular dataset programmatically (Table 3). Code to access a stream of GitHub releases could be written individually by each user, but this creates an unnecessary technological hurdle. To make it easier for users to access versioned data via code, we developed a new package for the R platform, as one of the most prominent platforms for data science [19].

Our package, called datastorr [22], facilitates access to releases of any evolving dataset hosted on GitHub (Fig. 1). Specifically, the datastorr package (i) contains the main code needed to interact with the GitHub API to retrieve versions of the dataset; and (ii) enables users to construct the shell of a second, dataset-specific R package, which can be distributed and used to access releases for a specific repository stored on GitHub. Using datastorr, a researcher can create and distribute a custom R package that facilitates access to their data with (very) minimal computational skills.

For example, datastorr has been used to build several packages (Table 2), including taxonlookup [24], which hosts data on the taxonomy of the world’s land plants [15]. The R package taxonlookup consists of only a few simple functions and associated help files that were automatically generated with datastorr. For a user, accessing a version of the data is a simple as typing a single line of code (Fig. 1). Accessing a different version of the data involves changing only the version number. From the user’s perspective, the existence of the taxonlookup and datastorr packages makes reproducing analyses using specific versions of the data [e.g., 15, 34] possible.

Using datastorr, dataset developers can set up their own R package to deliver versions of an evolving dataset simply by providing the following:

1. a GitHub repository name (e.g., “traitecoevo/taxonlookup”) where releases are stored;
2. the filename in the release that contains data;
3. the function used to load the data file into R.

Then as the dataset grows over time, the developers update the git repository and create a GitHub release with a new version number. All the releases are simultaneously available to any user, both point-and-click and programmatically.

The dataset-specific packages created by datastorr are designed to be computationally efficient and also work offline. Packages created by datastorr contain no actual data, only the rules for fetching the data. As such, the basic package structure is quick to install and takes up virtually no space on the user’s hard drive. The package functions by fetching each data version once (the first time it is requested) and then caching these files locally for future reuse. Moreover, users can store several versions of an evolving dataset on their computer and unambiguously access different versions with a single function.

## Discussion

The key issue we are dealing with in this article may be familiar to many readers: many datasets are constantly evolving and, despite tremendous advances in data sharing and associated technologies over the last decade, there is as yet little consensus about how to maintain and distribute multiple versions of an evolving dataset, especially for small research teams. While such teams could in principle create their own dynamic web interface, the technological hurdles, cost, and maintenance required are discouraging. Moreover, existing platforms for distributing data offer a limited set of features for the delivery of successive versions of an evolving dataset. This suggests that there is a need for an easy, cheap, and scalable solution for maintaining and distributing successive versions of an evolving dataset. By adopting open source and scalable practices from software development, we believe a workable system already largely exists. To aid this process, we created the datastorr package to deliver dataset versions directly into the R environment. The approach and package are already being used to deliver versions of several evolving datasets spanning a wide range of topics (Table 2). Moreover, because it builds off established and open source software and data science platforms (Table 1), the proposed system is already easy to deploy on a relatively large scale.

### Towards an ecosystem for evolving data

Our contribution here connects with a growing number of recommendations and technologies supporting the sharing and reuse of evolving data. Such contributions include community guidance on good practice in data curation [6, 8], data citation [7], and the FAIR principles for making datasets findable, accessible, interoperable, and reusable by both machines and humans [11]. In our proposed system, information about appropriate attribution for any dataset (whatever that is determined to be) should be made readily available, either on the landing page within GitHub or, even better, distributed as part of the versioned dataset itself. Similarly, datasets can be structured to make them follow the FAIR principles, to the extent possible. Notably, our workflow with the datastorr package demands machine access to datasets—a core focus for the FAIR principles. While our proposed workflow does not currently enhance discoverability of new datasets, this is a broad challenge faced by all data platforms and researchers.

While our package datastorr offers an easy way for users of the R ecosystem to directly access dataset version, users of other languages can also access the datasets. Moreover, packages similar to datastorr would ideally be developed to make accessing dataset versions as easy as it is with datastorr.

Within the R ecosystem, the datastorr package complements other approaches for creating and delivering datasets. One common approach used within R is to embed data directly within an R package, which can then be distributed via the Comprehensive R Archive Network [35]. Moreover, dedicated packages are being developed to assist dataset developers in creating data packages [36]. An advantage of this approach, compared to ours, is that the data are immediately available in the package (whereas our packages only contain instructions for fetching the data). This advantage however also brings limitations. Notably, datasets must be <5 MB, and only 1 version of a dataset package can be installed on any given machine at any one time. datastorr offers a viable approach for overcoming these limitations.

There are also many emerging or alternative technologies that offer other possible ways to implement a system for storing and distributing versions of an evolving dataset. Our solution currently emphasizes the platform GitHub, but similar functions could be achieved via other git hosts such as bitbucket.org [37] and gitlab.com [38]. Git repositories can also be extended to accommodate larger files using such features as Git-Large File Storage [39] or Git-Annex [40]. More fundamentally, there are emerging alternatives for version control specifically designed for data, such as the datproject.org [41], and other new platforms for distributing data, such as the Comprehensive Knowledge Archive Network (CKAN) [42] and Open Knowledge International (OKFN) [43].

The key here is not the specific technology but rather the concept of creating, maintaining, and distributing versions of an evolving dataset, which can be achieved with all of these approaches. Indeed, as with every technology the best available approach is certain to evolve, especially as emerging technologies facilitate even better delivery of data in the future.

### Further advantages and extensions

A central feature of the proposed system is that data are maintained on the web. This has 2 main benefits: first, it provides a platform for multiple data contributors to sync their files and correspond about changes in the dataset, and second, it allows for hosting of a stream of data releases for distribution (Fig. 1). Web platforms thus act as a central point for the collection, curation, and distribution of the data. Additionally, one of the greatest benefits of using web platforms like GitHub for development of both software and data has been the way they encourage contributions from multiple individuals working simultaneously—including from people outside the initial group of project participants [9, 44]. Multiple developers can make changes to different parts of the code (or, in our case, data) and the git system will integrate these together or, when needed, flag where there are conflicts that need to be resolved. The proposed system of data delivery thus has the added benefit of facilitating seamless and transparent collaboration among research groups in the construction and maintenance of datasets.

An important concern for any data delivery system is the stability and reliability of the system. In the short term, users want minimal downtime, high speed, and seamless operation. As one of the largest companies hosting computer source code, GitHub provides exceptional performance in this regard—certainly as good or better than nearly any system scientists might build themselves. Thus, short-term concerns of reliable and fast performance are almost guaranteed.

In the long term, scientists want their datasets, software, and papers to preserved and remain accessible. While our proposed system for data delivery does not guarantee long-term preservation, users can also choose to automatically archive data versions released on GitHub version in one of several traditional data archives, with a DOI minted for each release. Currently, both Zenodo and FigShare each integrate with GitHub for archiving of material hosted there. Ideally, tools like datastorr would also be developed to pull versions from these archives too.

## Availability of supporting data and materials

Snapshots of the code and example file are archived in the *GigaScience* GigaDB repository [45].

- Project name: datastorr
- Project home page: github.com/ropenscilabs/datastorr
- Operating system(s): platform independent
- Programming language: R
- License: MIT

- Project name: datastorr example
- Project home page: github.com/richfitz/datastorr.example
- Operating system(s): platform independent
- Programming language: R
- License: MIT

- Project name: taxonlookup
- Project home page: github.com/traitecoevo/taxonlookup
- Operating system(s): platform independent
- Programming language: R
- License: MIT

## Abbreviations

API: application programming interface; CKAN: Comprehensive Knowledge Archive Network; DOI: Digital Object Identifier; FAIR: findable, accessible, interoperable, reusable; Gb: gigabyte; Mb: megabyte; OKFN: Open Knowledge International.

## Competing interests

The authors declare that they have no competing interests.

## Funding

D.S.F. was funded by the Australian Research Council (FT160100113). M.W.P. was funded by an NSERC Discovery Grant (RGPIN-2017-04590).

## Authors' contributions

R.F. developed the datastorr package. All authors discussed the concepts and wrote the manuscript.

## Supplementary Material

GIGA-D-18-00005_Original_Submission.pdf

GIGA-D-18-00005_Revision_1.pdf

GIGA-D-18-00005_Revision_2.pdf

GIGA-D-18-00005_Revision_3.pdf

Response_to_Reviewer_Comments_Original_Submission.pdf

Response_to_Reviewer_Comments_Revision_1.pdf

Response_to_Reviewer_Comments_Revision_2.pdf

Reviewer_1_Report_Original_Submission -- Daniel Katz 1/14/2018 Reviewed

Reviewer_1_Report_Revision_1 -- Daniel Katz 10/30/2018 Reviewed

Reviewer_2_Report_Original_Submission -- Alex Ball 3/20/2018 Reviewed

Reviewer_3_Report_Original_Submission -- Greg Janée 3/26/2018 Reviewed
