# Supplementary material for: Datastorr: a workflow and package for delivering successive versions of 'evolving data' directly into R
Source: Gigascience. 2019 May 1;8(5):giz035. doi: 10.1093/gigascience/giz035 (PMC6506717; doi:10.1093/gigascience/giz035)

GigaScience

Versioned data: why it is needed and how it can be achieved (easily and cheaply)

--Manuscript Draft--

**Manuscript Number:**	GIGA-D-18-00005
**Full Title:**	Versioned data: why it is needed and how it can be achieved (easily and cheaply)
**Article Type:**	Technical Note
**Funding Information:**	
**Abstract:**	The sharing and re-use of data has become a cornerstone of modern science. Multiple platforms now allow quick and easy data sharing. So far, however, data publishing models have not accommodated on-going scientific improvements in data: for many problems, datasets continue to grow with time -- more records are added, errors fixed, and new data structures are created. In other words, datasets, like scientific knowledge, advance with time. We therefore suggest that many datasets would be usefully published as a series of versions, with a simple naming system to allow users to perceive the type of change between versions. In this article, we argue for adopting the paradigm and processes for versioned data, analogous to software versioning. We also introduce a system called Versioned Data Delivery and present tools for creating, archiving, and distributing versioned data easily, quickly, and cheaply. These new tools allow for individual research groups to shift from a static model of data curation to a dynamic and versioned model that more naturally matches the scientific process.
**Corresponding Author:**	Daniel S. Falster, PhD<br><br>AUSTRALIA
**Corresponding Author Secondary Information:**	
**Corresponding Author's Institution:**	
**Corresponding Author's Secondary Institution:**	
**First Author:**	Matthew Pennell
**First Author Secondary Information:**	
**Order of Authors:**	Matthew Pennell
	Daniel Falster
	Richard FitzJohn
	William Cornwell
**Order of Authors Secondary Information:**	
**Opposed Reviewers:**	
**Additional Information:**	
**Question**	**Response**
Are you submitting this manuscript to a special series or article collection?	No
**Experimental design and statistics**<br><br>Full details of the experimental design and statistical methods used should be given in the Methods section, as detailed in our Minimum Standards Reporting Checklist. Information essential to interpreting the data presented should be made available	Yes

Powered by Editorial Manager® and ProduXion Manager® from Aries Systems Corporation

in the figure legends.<br><br>Have you included all the information requested in your manuscript?	
**Resources**<br><br>A description of all resources used, including antibodies, cell lines, animals and software tools, with enough information to allow them to be uniquely identified, should be included in the Methods section. Authors are strongly encouraged to cite Research Resource Identifiers (RRIDs) for antibodies, model organisms and tools, where possible.<br><br>Have you included the information requested as detailed in our Minimum Standards Reporting Checklist?	Yes
**Availability of data and materials**<br><br>All datasets and code on which the conclusions of the paper rely must be either included in your submission or deposited in publicly available repositories (where available and ethically appropriate), referencing such data using a unique identifier in the references and in the “Availability of Data and Materials” section of your manuscript.<br><br>Have you have met the above requirement as detailed in our Minimum Standards Reporting Checklist?	Yes

Manuscript Click here to download Manuscript falster_ms_gigascience.pdf

OXFORD (GIGA)^n SCIENCE

GigaScience, 2018, 1–6

doi: xx.xxxx/xxxx
Manuscript in Preparation
Technical Note

TECHNICAL NOTE

Versioned data: why it is needed and how it can be achieved (easily and cheaply)

Daniel S. Falster[1,*], Richard G. FitzJohn[2], Matthew W. Pennell[3] and William K. Cornwell[1]

[1]Evolution & Ecology Research Centre, School of Biological, Earth and Environmental Sciences, University of New South Wales, Sydney NSW 2052, Australia and [2]School of Public Health, Imperial College, London SW7 2AZ United Kingdom and [3]Department of Zoology and Biodiversity Research Centre, University of British Columbia, Vancouver B.C. V6T 1Z4, Canada

[*]daniel.falster@unsw.edu.au

Abstract

The sharing and re-use of data has become a cornerstone of modern science. Multiple platforms now allow quick and easy data sharing. So far, however, data publishing models have not accommodated on-going scientific improvements in data: for many problems, datasets continue to grow with time – more records are added, errors fixed, and new data structures are created. In other words, datasets, like scientific knowledge, advance with time. We therefore suggest that many datasets would be usefully published as a series of versions, with a simple naming system to allow users to perceive the type of change between versions. In this article, we argue for adopting the paradigm and processes for versioned data, analogous to software versioning. We also introduce a system called Versioned Data Delivery and present tools for creating, archiving, and distributing versioned data easily, quickly, and cheaply. These new tools allow for individual research groups to shift from a static model of data curation to a dynamic and versioned model that more naturally matches the scientific process.

Key words: Data sharing; Version control; Semantic versioning; Meta-analysis

Introduction

Publications of quality datasets are now considered a first-class scientific products. Increasingly, funding bodies, publishers, and scientific social norms are recognizing the value of sharing data, including as standalone products without any accompanying analyses [1, 2, 3, 4, 5]. Datasets are now routinely archived as part of the publishing process. Moreover, increasing numbers of standalone "Data papers" (or descriptors) have been appearing in standard domain-level journals, as well as specialised data journals. Yet, while the last decade has witnessed a rapid and exciting change in attitudes towards data sharing and publishing, the scientific community is still grappling with how to effectively disseminate and manage open-source datasets [1, 6, 7, 8, 4, 9]. In particular, the current model for publishing data has not yet embraced the idea that many datasets are designed to answer scientific questions that extend beyond the scope of a single empirical paper. These datasets are constantly evolving, i.e. they are never "finished", and may be the subject of repeated analysis or meta-analysis.

Many of the high-quality datasets published in scientific journals are likely to be living entities, meaning the state-of-the-art (or "canonical") version of the dataset will change through time. Typical changes include: the adding new data, improving the quality of existing data, integrating with other datasets, or re-structuring the dataset content. For example, a dataset on a biological organisms might be expanded through the addition of new records or improved through the correction of spelling mistakes in taxonomic names. As research around a data product grows, there might be many such additions. Ideally, any changes to a dataset would become immediately available to others, while past versions of the dataset would remain

Compiled on: December 15, 2017.
Draft manuscript prepared by the author.

Key Points

- Datasets are often being expanded and improved; yet current publishing does not accommodate multiple dataset versions.
- We present a Version Data Delivery system, for updating and distributing versioned data.
- Using semantic versioning to label versions of dataset conveys helps users identify the type of change that has occurred.

accessible to ensure previous works can be reproduced.

Yet, current models for publishing datasets do not facilitate distributing successive versions of a dataset. The most common current working model for publishing scientific datasets is to archive them in a variety of open-source data repositories where the data is attributed a Digital Object Identifier (DOI) – such as `DataDryad`, `Figshare`, or `Zenodo`. Publishing involves a single deposition. Once published, datasets are immutable. We refer to this type of data delivery as being "Static" (Table 1). In the Static model, published datasets exist as a singular snapshot taken at the time the data descriptor was published. While it might only require a small amount of work to update the dataset, if there is no way to distribute the updated dataset, that work has to be re-done by every user of that dataset. Moreover, those required changes add steps between the canonical dataset and any analysis using it, making reproducibility of the new analysis more difficult than it needs to be.

Many research groups solve the issue of versioning data internally in a variety of ad-hoc and suboptimal ways. A common solution is to email around the latest version with version numbers or dates appended to the filename. Another approach is to repeat corrections or updates that have been made elsewhere. Alas, these practices are both inefficient and a barrier to reproducibility, as the dataset used in a future publications may differ to the version previously made available on-line. There is therefore a strong need for an easy way to distribute updates to a dataset after its initial publication, along with notes on what has changed and why since the previous version.

In this article we 1) Introduce the concept of Versioned Data Delivery (VDD); 2) Outline how emerging technologies in data science (Table 2) can be used to help researchers maintain, distribute, and access small-to-medium sized datasets; and 3) Introduce a new R package called `datastorr`, a proof-of-concept implementation of a VDD system. The issue of updating and expanding published data has already been addressed in large centralised repositories like genetic sequences (`GenBank`) or species location data, where new data can be added and there exist abilities to correct errors in existing records (e.g. by adding multiple version of a genetic sequence). Yet these "Dynamic" web databases (Table 1), require a level of infrastructure that is beyond most research groups. Our focus here is on the wide range of datasets, such as those appearing in this journal, that are not covered by these repositories. In most of these cases, the data collected will not be "big" but rather small-to-medium sized. Such data support important research projects on particular scientific questions, but are not, in most cases, general enough to warrant custom infrastructure. Further, while we emphasize particular technologies in our implementation, the principles are general and could easily be ported to other platforms.

A lightweight, cheap, and scalable workflow for delivering versioned data

In brief, the VDD workflow we present borrows best-practices for software development [10] and applies them to the challenge of maintaining and distributing versioned data. Software developers maintain a core set of code which produces a binary executable file that can be installed on a users local computer. With a VDD system, data developers similarly maintain a core set of files (the "code"), which produce an organised dataset that can be "installed" on a user's local computer. In either the development of software or data, successive versions (called "releases") are distributed over time. The similarity in workflow between software and data then allows us to deploy the same technological platforms that are used in software development, for the development and distribution of data. Importantly, our VDD systems uses well-established tools (Table 2), ensuring high-level performance and stability.

The core technologies used are summarised in Table 2 and described further below. Several groups will interact with the VDD system, including dataset *maintainers*, *contributors*, and *users*. The requirements of each are outlined in Table 3.

Version control

Version control, primarily an open-source variety called `git`, has become ubiquitous in software development. In practice, version control tracks line-by-line changes in text files and creates and maintains a history of those changes. Increasingly version control has been applied to scientific code and data management, especially for small-to-medium sized datasets [11, 8, 7]. `git` is attractive for data management because it tracks all changes in monitored files, provided these are saved in text format (e.g. ".csv", ".tsv", ".txt"). The history is visible to anyone interacting with the repository. It also allows users to annotate changes ("commits") with informative messages detailing the rationale for those changes. In it's present form, `git` can handle individual data files at least up to 100MB, which includes a large fraction of scientific cases.

As a general strategy for tracking a dataset under version control with git, we recommend:

i. Dataset *maintainers* establish a separate `git` repository for any dataset to be distributed.
ii. Saving all files as plain text, so that `git` can identify line-by-line changes. For example, you might maintain tabular data as a "csv".
iii. Saving data in their rawest form. In some datasets you might only have a single file. Others may have may files that get manipulated or combined in some way to produce a unified product.
iv. Including in the `git` repository any code needed to manipulate or compile the raw data files into the final dataset. For example, you might combine many independent datasets into one unified dataset.
v. Documenting any change in the dataset as a "commit" into the `git` repository logs, with informative commit messages outlining why a particular change was made.

Hosting and distributing versioned data

Datasets stored under version control via `git` reach their real potential when hosted at a suitable internet hosting service

Table 1. Alternative frameworks for distributing data.

Feature	Static datasets	Web databases	Versioned Data Delivery
Platform (e.g.)	datadryad.org	coraltraits.org	github.com
User access	Web browser	Web browser	Web browser or R
Ease of setup	Very easy	Hard	Easy
Data size	Up to several Gb	Small-Very large	Up to 1Gb
Cost	Varies	Varies	Free
Bandwidth	Managed by provider	Pro rata	Managed by `GitHub`
Maintainer skills	None	High	R + `git`
User skills	Web browsing	Web browsing	Web browsing or R
Versioning	None	Hard	Easy
DOI minting	Automatic	Manual	Automatic

[11, 8]. Here we focus on the platform `GitHub` (Table 2), but similar functions could be achieved via other providers such as bitbucket.org and gitlab.com. Hosting of a `git` repository enables dataset *maintainers* to connect with other potential *contributors* and also *users* (Fig. 2). These platforms are designed to work with `git` repositories, and thus offer many helpful features, such as ability to record issues, host documentation, or review edits over time.

Of particular interest for current purposes, is `GitHub`'s ability to host a stream of "releases" from the dataset, alongside the `git` repository containing all the rawfiles. Each release is linked to a specific commit in the `git` repository history and occur at points where the dataset *maintainer* decided to generate a new version of the data for distribution. While *users* could in principle download the entire `git` repository, actually all they want are the releases.

Deciding when to make a new release is at the discretion of the dataset *maintainer*. In practice, one makes fewer releases than one does commits into the `git` repository, though there is nothing stopping *maintainers* from releasing a new version for every commit. The flexibility here allows *maintainers* to do internal work between releases and only release the data to users when the revision represents a clear improvement on the previous release.

Another important consideration is that websites like `GitHub` naturally cater for two types of data users accessing the data: those that interact with the data via point and click downloading, and those that use programmatic interaction (Fig. 2, Table 3). Specifically, `GitHub` releases can be downloaded directly by users or accessed programmatically via the `GitHub` API.

Semantic versioning

To realize the full benefits of a versioned controlled dataset, users should be able to easily intuit the types of changes that have occurred among versions. Since software development has effectively already dealt with almost identical; problem in the labelling of software releases, we suggest there is benefit in adopting the best-practices from that field.

Specifically, we suggest applying the theory of "semantic versioning", developed for software distribution (semver.org), to successive releases of a dataset. Semantic versioning uses tri-digit notation of the form "X.Y.Z" for successive versions, where X, Y, and Z are non-negative integers. For example, version "1.0.0". Changes in the version number then signal the type of changes that occurred in the dataset (Fig 1). Applying semantic versioning to data, a change from:

- **1.0.0** → **1.0.1**: implies a "correction" (or "patch"), for example a small error correction which is unlikely to break, or change in a substantial way, a users' analyses, although there may be minor changes in the results.
- **1.0.0** → **1.1.0**: implies a "minor" enhancement, for example adding a new study to a meta-analysis dataset (while otherwise maintaining the same dataset structure); this change is large enough that in the view of the dataset curator it might change the results of an analysis done with the prior version and so warrants closer inspection.
- **1.0.0** → **2.0.0**: implies a "major" revision, for example improving the entire structure of the dataset or adding new columns. These changes are likely to change the results of most users' analyses or to break code that was written to work on the previous version of the dataset.

Any user of a dataset where releases were tagged using semantic versioning, would immediately know the types of change that might have occurred when requesting different versions and set their there expectations accordingly.

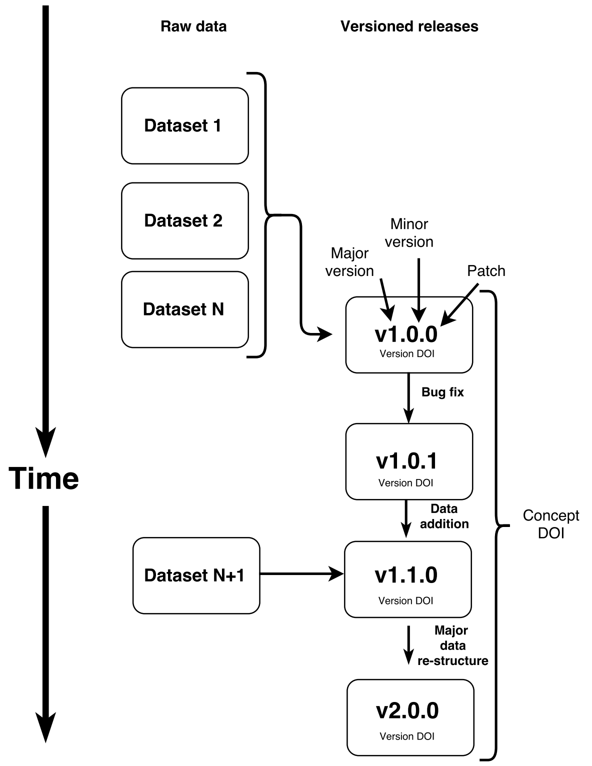

Figure 1. Semantic versioning allows users to anticipate the types of changes that have occurred between successive versions of a dataset. **a)** 3 numbers indicate type of change. **b)** A typical release stream.

Table 2. Overview of technologies used to maintain, store, and distribute the versioned data as described in this paper.

Technology	Description
API	An Application Programming Interface provides a set of protocols for exchanging information.
`datastorr`	R package used to fetch versioned releases from `GitHub`.
DOI	Digital object identifier, which refers a user to a single published object.
`git`	Open source version control system used for tracking progressive changes in a set of text files, typically computer code but also data.
github.com	A commercial web platform for sharing, visualising, and managing 'git' repositories. Includes ability to browse the 'history', 'issue' tracking, and ability to create 'releases'.
`R`	Open source statistical and data processing language.

The `datastorr` package and dataset-specific `R` packages

To aid reproducibility and efficient usage, many users will want access to all versions of any particular dataset programmatically (Table 3). Code to access a stream of `GitHub` releases could be written individually by each user, but this creates an unnecessary technological hurdle. To make it easier for users to access versioned data via code, we offer a novel implementation of VDD focussing on the `R` platform, as one of the most prominent platforms for data science [12].

Here we introduce a dedicated R package, called `datastorr` (github.com/ropenscilabs/datastorr), that facilitates access to releases of any versioned data hosted on `GitHub` (Fig. 2). Specifically, the `datastorr` package: 1)Constructs the shell of a second, dataset-specific R package, which is used to access releases from a specific repository stored on `GitHub`; and 2) Contains the main code needed to interact with the `GitHub` API to retrieve versions of the dataset. Using `datastorr`, a researcher can create and distribute a custom R package that facilitates access to their data with (very) minimal computational skills.

For example, `datastorr` has been used to build several packages (Table 4), including `baad.data` (github.com/traitecoevo/baad.data), which is an interface to the Biomass and Allometry Database [13] stored at github.com/dfalster/baad. The R package `baad.data` consists of only a few simple functions and associated help files, that were automatically generated with `datastorr`. For a user, accessing a version of the data is a simple as typing a single line of code (Fig. 2). Accessing a different version of the data involves changing only the version number.

From the users perspective, the existence of the `baad.data` and `datastorr` packages makes reproducing analyses using specific versions of the data possible [14, 15].

Using `datastorr`, dataset *maintainers* can set up their own R package to deliver there versioned dataset simply by providing:

i. a `GitHub` repository name (e.g., "traitecoevo/baad.data") where releases are stored;
ii. the filename in the release that contains data;
iii. the function used to load the data file into `R`.

A full tutorial explaining precisely how to set this up is available at github.com/ropenscilabs/datastorr.

Then as the dataset grows over time, the *maintainers* update the `git` repo and create a `GitHub` release with a new version number. All the releases are simultaneously available to any user, both point-and-click and programmatically.

The dataset-specific packages created by `datastorr` are designed to be computationally efficient and also work offline. Packages created by `datastorr` contain no actual data, only the rules for fetching the data. As such, the basic package structure is quick to install and takes up virtually no space on the user's hard-drive. The package functions by fetching each data version once (the first time it is requested), and then caching these files locally for future reuse. Moreover, *users* can store several versions of a dataset on their computer and unambiguously access different versions with single function.

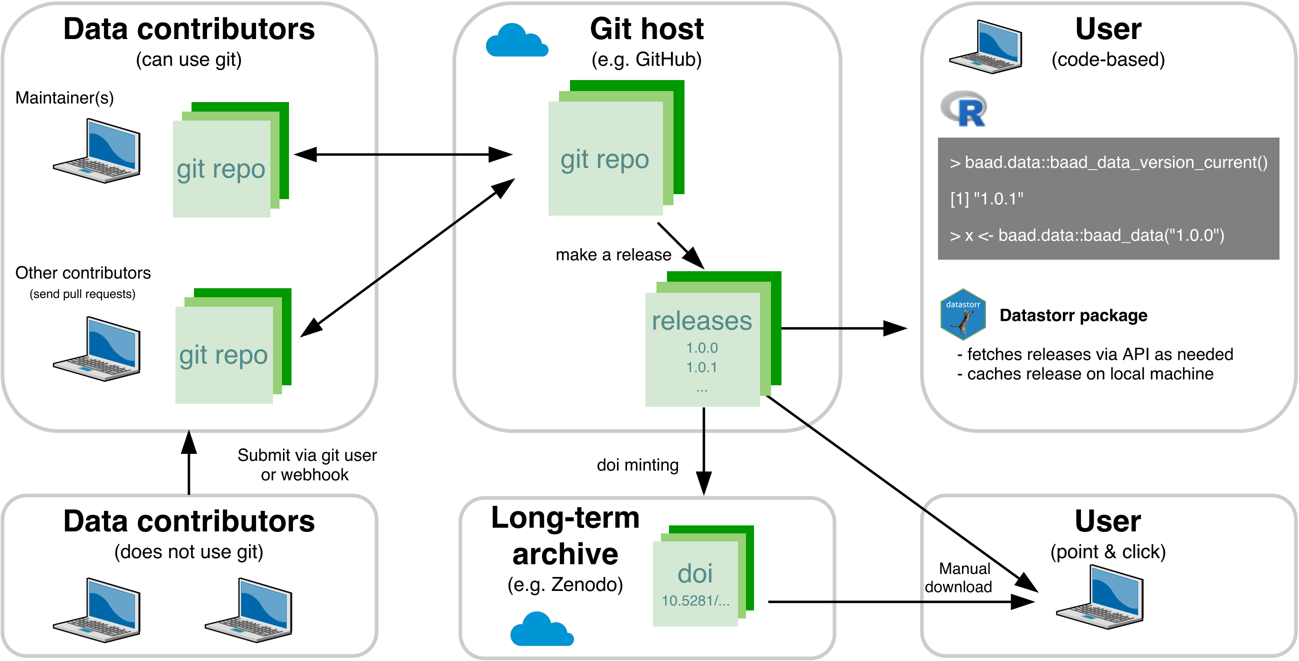

Figure 2. Overview of the different users and technologies involved in distributing a versioned dataset via `datastorr`.

Table 3. Groups of users interacting with the Versioned Data Delivery system described and their requirements.

Group	Goal	Requirements
Maintainer	Create and distribute versioned datasets	Low technical overhead<br>Easy workflow for releasing new versions<br>Long term preservation<br>Crowd-source error checking and contributions<br>Low initial and ongoing cost and maintenance
Contributor	Contribute to future versions of a dataset	Add new data<br>Report errors in existing data
Users (all)	Easy access to releases from a versioned dataset	Introduction & overview<br>Long term stability<br>Clear path for users to become contributors
Users (programmatic)	Reproduce products using specific versions of a dataset	Programmatic access to releases<br>Easy installation<br>Long term stability

Discussion

The main issue we have identified in this article will not come as news to many readers: datasets are constantly improving and, despite tremendous advances in data sharing and associated technologies over the last decade, there is little consensus about how to handle dataset updates. Some specific solutions for versioning already exist, such as those designed for genetic sequence data (e.g., GenBank; see www.ncbi.nlm.nih.gov/genbank/sequenceids/). But so far, versioning solutions have only been developed for large dynamic databases managed by well-funded institutions or consortia and involving simple standardised data types. While individual research groups working with a specialized type of data could in principle create their own dynamic web interface, the technological hurdles, cost and maintenance required (Table 1) will be discouraging for most. This suggests there is a substantial need for an easy, cheap, and scalable solution for serving versioned data. By adopting best practices from software engineering, we believe a workable system now exists. Moreover, we created the datastorr package to make such a VDD system easy to implement for R users. As it builds off established and open source software and data science platforms, the proposed system is already easy to deploy on relatively large scale.

A central feature of the proposed VDD system is that data are maintained in the cloud. This has two main benefits: first, it provides a platform for multiple data contributors to sync their files and correspond about changes in the dataset, and second, it allows for hosting of a stream of data releases for distribution (Fig. 2). Cloud systems thus act as a central point for the collection, curation, and distribution of the data. Additionally, one of the greatest benefits of using cloud-based tools like GitHub for development of software and data has been the way they encourage contributions from multiple individuals working simultaneously — including from people from outside the initial group of project participants [17, 8]. Multiple users can make changes to different parts of the code (or in our case, data) and the git system will integrate these together (if that is possible) or, when needed, flag where there are conflicts that need to be resolved. Adopting a VDD system thus has the added benefit of facilitating seamless and transparent collaboration among research groups in the construction and maintenance of datasets.

An important concern for any data delivery system is the stability and reliability of the system. In the short term, users want minimal downtime, high speed, and seamless operation. As one of the largest companies hosting computer source code, GitHub provides exceptional performance in this regard – certainly as good or better than nearly any system scientists might build themselves. In the long term, scientists want their datasets, software, and papers to preserved and remain accessible. To enhance long-term stability of data-versions released on GitHub, users can also choose to automatically archive every version in one of several traditional data archives, with a DOI (Digital Object Identifier) minted for each release. Currently, both Zenodo (zenodo.org) and FigShare figshare.com each integrate with GitHub for archiving of material hosted there.

The design choices we made in datastorr represent only one of many possible ways to adopt a VDD system; we can imagine many alternative models for interacting with versioned data stored in the cloud. The key is not the specific technology, but rather the concept of creating, maintaining, and distributing versioned data. Indeed, as with every technology the best available approach is certain to evolve, especially as merging technologies — such as CKAN, OKFN or Git-LFS — facilitate even better VDD systems in the future. There are some really exciting ideas that are currently under development which will make versioning systems faster computationally, smaller in file size, flexible with respect to data structures, and thus allow for larger datasets to be versioned efficiently [18, 19].

In short, many of the key roadblocks preventing a switch from a static to a dynamic data world were technological: in the past, it took great deal of money and expertise to set up and maintain a VDD system. By adopting best practices from software engineering, we believe a relatively easy, cheap, and scalable solution for serving versioned data now exists.

Availability of source code and requirements

- Project name: datastorr
- Project home page: github.com/ropenscilabs/datastorr.
- Operating system(s): Platform independent
- Programming language: R
- License: MIT

Declarations

Table 4. Example datasets using the Versioned Data Delivery system described in this paper.

GitHub repo	R package	Description
dfalster/baad	baad.data	The Biomass And Allometry Database provides data on the size dimensions of plants for many species, compiled from multiple scientific papers [13].
traitecoevo/taxonlookup	taxonlookup	Provides a taxonomic lookup table for land plants [16].

List of abbreviations

API = An Application Programming Interface provides a set of protocols for exchanging information. DOI = Digital Object Identifier. VDD = Versioned Data Delivery

Ethical Approval

Not applicable.

Consent for publication

Not applicable.

Competing Interests

The author(s) declare that they have no competing interests.

Funding

DSF was funded by the Australian Research Council. MWP was funded by a NSERC Discovery Grant.

Author's Contributions

RF developed the datastorr package. All authors discussed the concepts and wrote the paper.

Acknowledgements

We thank D Noble for comments on an earlier draft and C Boettiger for helpful discussions.

References

1. Whitlock MC. Data Archiving in Ecology and Evolution: Best Practices. Trends in Ecology & Evolution 2011;26:61–65.
2. Fairbairn DJ. The Advent of Mandatory Data Archiving. Evolution 2011;65:1–2.
3. Piwowar HA, Vision TJ, Whitlock MC. Data Archiving Is a Good Investment. Nature 2011;473:285–285.
4. Van Noorden R. Data-Sharing: Everything on Display. Nature 2013;500:243–245.
5. Gibney E, Van Noorden R, Scientists Losing Data at a Rapid Rate; 2013. Nature news. doi: 10.1038/nature.2013.14416.
6. Goodman A, Pepe A, Blocker AW, Borgman CL, Cranmer K, Crosas M, et al. Ten Simple Rules for the Care and Feeding of Scientific Data. PLoS Comput Biol 2014;10:e1003542.
7. Lowndes JSS, Best BD, Scarborough C, Afflerbach JC, Frazier MR, O'Hara CC, et al. Our Path to Better Science in Less Time Using Open Data Science Tools. Nature Ecology & Evolution 2017;1:0160.
8. Perkel J. Democratic Databases: Science on GitHub. Nature 2016;538:127–128.
9. Kratz JE, Strasser C. Making Data Count. Scientific Data 2015;2:sdata201539.
10. Perez-Riverol Y, Gatto L, Wang R, Sachsenberg T, Uszkoreit J, da Veiga Leprevost F, et al. Ten Simple Rules for Taking Advantage of Git and GitHub. PLOS Comput Biol 2016;12:e1004947.
11. Ram K. Git Can Facilitate Greater Reproducibility and Increased Transparency in Science. Source Code for Biology and Medicine 2013;8:7.
12. R Core Team. R: a language and environment for statistical computing. R Foundation for Statistical Computing, Vienna, Austria; 2017, http://www.R-project.org/.
13. Falster DS, Duursma RA, Ishihara MI, Barneche DR, FitzJohn RG, Vårhammar A, et al. BAAD: a Biomass And Allometry Database for woody plants. Ecology 2015;96:1445.
14. Duursma RA, Falster DS. Leaf Mass per Area, Not Total Leaf Area, Drives Differences in above-Ground Biomass Distribution among Woody Plant Functional Types. New Phytologist 2016;212:368–376.
15. Falster DS, Duursma RA, FitzJohn RG. Trajectories: how functional traits influence plant growth and shade tolerance across the life-cycle. bioRxiv 2016;p. 083451.
16. Pennell MW, FitzJohn RG, Cornwell WK. A simple approach for maximizing the overlap of phylogenetic and comparative data. Methods in Ecology and Evolution 2015;7:751–758.
17. Rogers M, The Github Revolution: Why We're All in Open Source Now; 2013. https://www.wired.com/2013/03/github/.
18. Fli, git for data; 2017. Accessed: 2017-08-03. http://clusterhq.com/fli/introduction/.
19. Dat, The distributed data sharing tool; 2017. Accessed: 2017-08-03. https://datproject.org/.

Figure_1

Click here to download Figure Figure1.pdf

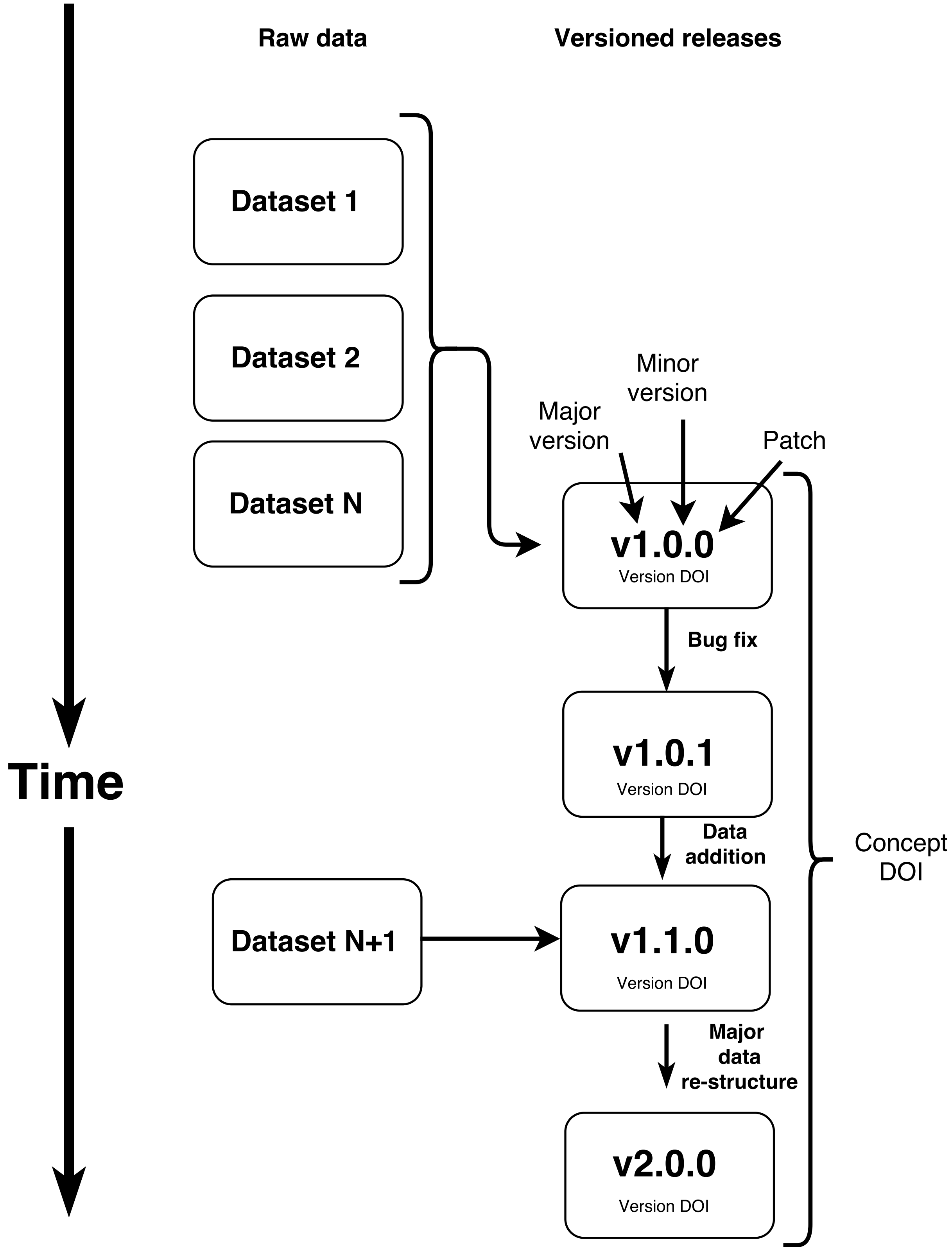

Figure_2

Click here to download Figure Figure2.pdf

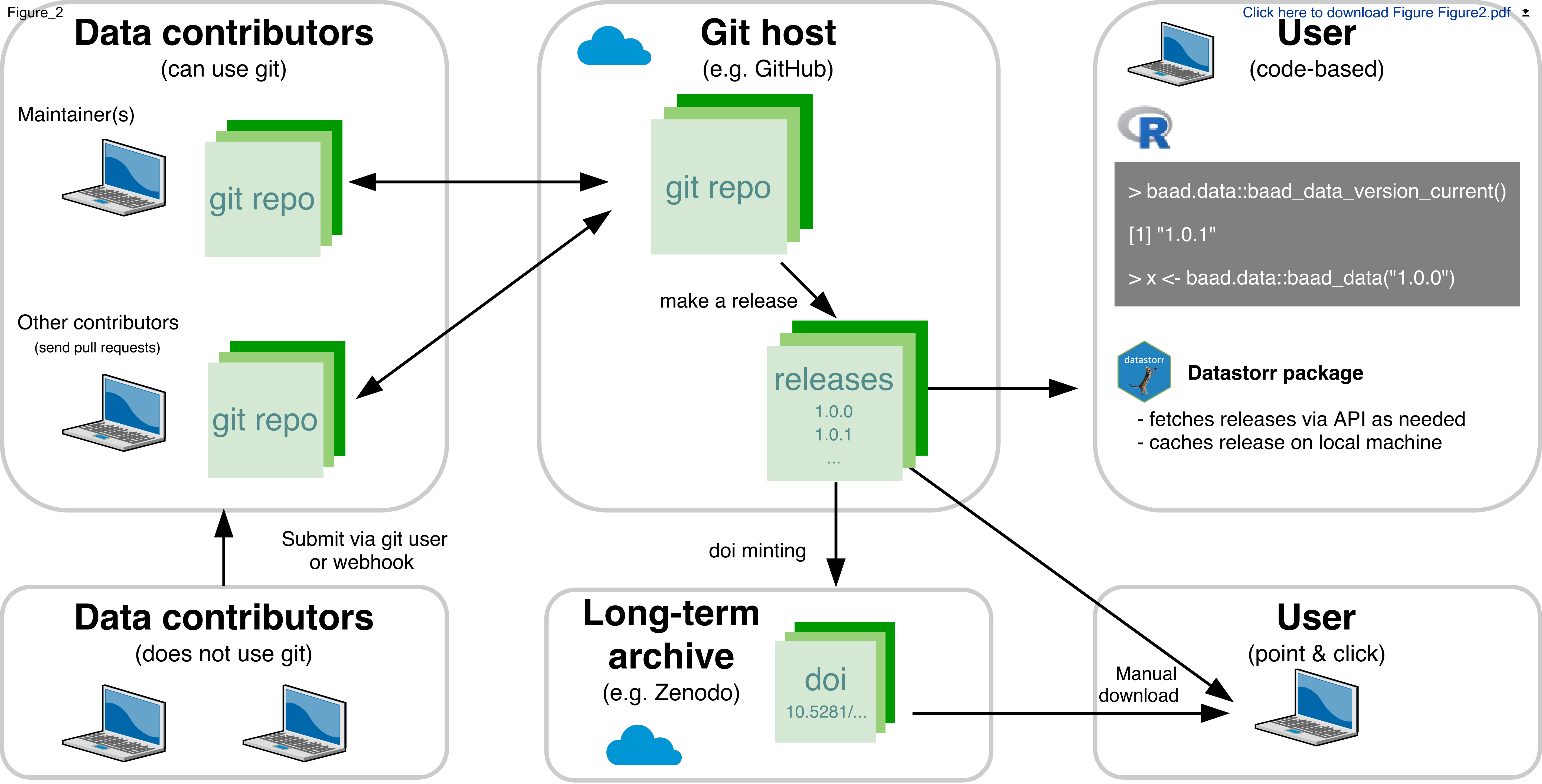

Supplement: GIGA-D-18-00005_Original_Submission.pdf [file giz035_giga-d-18-00005_original_submission.pdf]
