# Supplementary material for: Datastorr: a workflow and package for delivering successive versions of 'evolving data' directly into R
Source: Gigascience. 2019 May 1;8(5):giz035. doi: 10.1093/gigascience/giz035 (PMC6506717; doi:10.1093/gigascience/giz035)

GigaScience

Datastorr: a workflow and package for delivering successive versions of "evolving data" directly into R

--Manuscript Draft--

**Manuscript Number:**	GIGA-D-18-00005R1
**Full Title:**	Datastorr: a workflow and package for delivering successive versions of "evolving data" directly into R
**Article Type:**	Technical Note
**Funding Information:**	
**Abstract:**	The sharing and re-use of data has become a cornerstone of modern science. Multiple platforms now allow quick and easy data sharing. So far, however, data publishing offers limited functions for interacting with evolving datasets - those that continue to grow with time as more records are added, errors fixed, and new data structures are created. In this article, we describe a workflow for maintaining and distributing successive versions of an evolving dataset, allowing users to retrieve and load different versions directly into the R platform. Our workflow utilises tools and platforms used for development and distribution of successive versions of a open source software, including version control, GitHub and semantic versioning, and applies these to the analogous process of developing successive versions of an open source dataset. Moreover, we argue that this model allows for individual research groups to achieve a dynamic and versioned model of data delivery at no cost.
**Corresponding Author:**	Daniel S. Falster, PhD<br><br>AUSTRALIA
**Corresponding Author Secondary Information:**	
**Corresponding Author's Institution:**	
**Corresponding Author's Secondary Institution:**	
**First Author:**	Daniel Falster
**First Author Secondary Information:**	
**Order of Authors:**	Daniel Falster
	Richard FitzJohn
	Matthew Pennell
	William Cornwell
**Order of Authors Secondary Information:**	
**Response to Reviewers:**	Comments from Reviewer #1:<br><br>I think the topic of this paper is important, and the work done here is valuable. However, the paper as written is a bit muddled currently, and is not really well set in the context of related work.<br><br>> Thank you for the constructive review. All reviewers commented that the paper should be more focussed, and also needed stronger links to related work. We have followed these suggestions.<br><br>The specific foci of the paper and some comments on them are:<br><br>1. datasets often need version - this is fine<br><br>> Agreed.

Powered by Editorial Manager® and ProduXion Manager® from Aries Systems Corporation

2. version numbers - the general idea is fine, but the information about DOIs is a bit unclear. Figure 1 shows a concept DOI. How is this generated? How does it know about the individual version DOIs? Is this using RelationType metadata? Or adding information within the DOI name itself? And how will these DOIs be used? Would a person using a version of a data set cite both the version's DOI and the concept's DOI? The paper does not contain enough discussion about DOIs and their usage.

> Minting of DOI's is an optional extra in our workflow and does not impact on the functioning of our core workflow. We tried to make this clearer in the revised text, by
Highlighting "minting DOI" as an optional in the figure showing the overall workflow.
Removing DOIs from figure about semantic versioning altogether
Noting in the Discussion, that "Minting of DOIs" is an option extension.

3. the VDD system. It's also unclear if this paper is trying to make the case that this is needed, and that it can be provided by multiple packages, or if this is something that is just supported by the authors VDD system. In other words, is this is a general need and a general solution, or is this a specific solution using a specific tool? It seems to be doing a little of both, and it would be better to discuss the general problem first, then a general solution, then the specifics of the solution in VDD, to make this more clear. The penultimate paragraph of the paper does talk about this a little (and it also includes some related work), but it would be better to move this discussion to earlier in the paper. Related to this, it seems that the software discussed here is specific to R. How would a Python user use these ideas?

> As both reviewers note, versioning of datasets is not entirely new. We have therefore focussed our paper more explicitly on the particular workflow we are promoting, by:
In the introduction, framing versioning of datasets as an emerging approach with increasing attention (as opposed to an entirely novel idea)
Removing the previous Table 1, which offered a broader comparison of dataset publishing which did not fit with the narrower focus
Removing the phrase VDD (Versioned Data Delivery) as we have a specific rather than general tool
Including more discussion of related approaches (see Discussion), including other technologies and relevance for users of other languages (eg Python).

In addition:

This work should also be compared with http://git-annex.branchable.com, which shares many elements of this work. In fact, I would like to see a related work section, which could include table 1, more discussion, and potentially discussion of how other types of material with multiple versions are handled, including software and preprints/papers.

> As noted in the previous comment, we removed the previous Table 1. However, the revised discussion includes a broader discussion of related technologies, including git annex.

There could also be some discussion about fields in which data releases are common, and users are used to dealing with them. For example, the data releases for SDSS: http://www.sdss.org/dr14/ and http://www.sdss.org/collaboration/citing-sdss/

> Thanks for this great reference. We have included the SDSS in both the introduction and discussion.

I'm also a little surprised there is no mention of the FAIR data principles (https://doi.org/10.1038/sdata.2016.18) or the data citation principles (https://www.force11.org/group/joint-declaration-data-citation-principles-final) here.

> Agreed. These links are mentioned in the discussion, in the new section "Towards an ecosystem for evolving data"

Comments from Reviewer #2:

I think this is an interesting approach. I sense that the authors are being slightly disingenuous about the current state of affairs in order to make their solution more attractive, but there is no need of it.

> Thank you for the constructive review. During the process of review we have discovered multiple resources that indicate the general idea of versioning of data is not as new as we portrayed it to be. We have therefore revised the paper to focus more on our particular workflow, which assists with distributing versions of an evolving dataset.

Specific points:
- "The current model for publishing data has not yet embraced the idea that many datasets are designed to answer scientific questions that extend beyond the scope of a single empirical paper. These datasets are constantly evolving." While it may be true that all dynamic datasets are intended as a resource for multiple papers (otherwise why bother), the reverse is not true. There are plenty of static datasets that are provided explicitly as an ongoing resource for future research: major social surveys, outputs of astronomical missions, etc.

> Agreed. This sentence has been revised to read "In particular, in some areas, such as our own area of ecology and evolution, we are only starting to support the fact that some high-quality datasets may be evolving entities". Moreover, in the revised text we have tried to remove comments about general publishing of data and instead focus on instances where dataset developers want to distribute versions.

- "Yet, current models for publishing datasets do not facilitate distributing successive versions of a dataset." A similar point is made in Table 1. This is false. In our institutional data repository, based on Eprints, we can provide successive linked versions of a dataset. Both Zenodo and FigShare, later mentioned as providing the DOI minting and long-term archiving part of the proposed solution, can do likewise. I'm sure other platforms can as well.

> Agreed. The revises text explicitly acknowledges this fact (see Introduction)

I would much prefer it if the authors were clearer about what they are actually proposing here: a simple solution for research groups to maintain a dynamic dataset, and integration of this solution into R. It has been said that there is a difference between lowercase publication (public distribution) and uppercase Publication (guarantees of stability and long-term availability). This paper positions itself as addressing the latter but is in fact addressing the former, and indeed for Publication relies on the very solutions it dismisses in the introduction. As I say, there is no need of this. The solution is interesting enough to stand on its own merits, and I rather like it.

> Thank you. We have taken this suggestion on wholeheartedly and have reorganized the paper accordingly. The title, abstract, key points, introduction and discussion have all been reworked to focus on the key innovation, as identified by the reviewer: a simple solution for research groups to maintain a dynamic dataset, and integration of this solution into R

On a technical point, certain tricks can be used with Git to get meaningful diffs with some types of binary files (e.g. zipped XML file formats), but this is probably beyond the detail needed in this paper. The point about binary files is that using Git with them (in the absence of such tricks) does not give you any advantage over the regular linked snapshot approach used by data repository platforms.

> We added a minor note to this effect in the revised text (see section "version control").

Comments from Reviewer #3:
This paper describes a problem of importance and contemporary interest (versioning scientific datasets) and presents a practical and effective approach. Generally I like the authors' solution and presentation of the problem, and I think the paper makes a useful contribution.

	> Thank you for the constructive review.<br><br>The introduction (top of p. 2) is perhaps a bit overstated. Figshare (https://knowledge.figshare.com/articles/item/can-i-edit-or-delete-my-research-after-it-has-been-made-public), Dryad (https://datadryad.org//pages/policies#versioning), and Zenodo (http://help.zenodo.org/#versioning) do support versioning. Their approaches to versioning are perhaps not the most flexible (Dryad's is particularly limited of the three), but nevertheless it's not correct to say that datasets stored in those systems are entirely static. Perhaps the authors could revise the statement.<br><br>> Agreed. This text has been revised, as noted above in response to similar comments from R1 and R2.<br><br>My one criticism is that I don't think it is obvious how to translate semantic versioning from software to data. In the case of software, versioning benefits from an implicit sense of correctness. I say only a "sense," because the semantics of a piece of software are never formally specified except in very rare circumstances. Nevertheless, there is almost always an implied sense that the software is supposed to perform certain functions and give certain results, and people use the software and develop against the software making those implicit assumptions. This enables the common understanding that the fix of a bug in the software can usually be considered a patch change (1.0.0 -> 1.0.1) because the software's users were already expecting the software to behave that way, and so upgrading the software generally can't cause any harm. But in the case of data, what constitutes a patch change? A discovery that, say, the calibration of a sensor was mis-set, and a subsequent correction of that calibration, could be interpreted as a simple bug fix since it affects the "correctness" of the data: the calibration and the data were supposed to have the new values all along. And yet, the existence of that bug and its correction may be of huge importance: it may invalidate all previous uses of the data! The authors of this paper describe a patch change as "a small error correction which is unlikely to break, or change in a substantial way, a users' analyses". But which changes will break downstream analyses, and which will not? Developing an understanding between data producers and data consumers of what semantic versioning means for data, and how it is routinely applied, is perhaps outside the scope of this paper, but I think at minimum the authors should acknowledge this as an area of needed future work.<br><br>->This is a fantastic comment, which caused us to discuss our application of semantic versioning in more and revise our recommendations. The reviewer is right that the mapping from software to dataset is not perfect. In software the version numbers refer to changes in the public interface for the software (i.e. it's API). In our revised text we apply this idea of an Interface to the dataset and recommend version increments according to how they change this interface. We agree with the reviewer is absolutely right that dataset developers cannot guarantee an apparently "Minor" or "Patch increment won't have non-trivial impacts on prior work. We now acknowledge and discuss this.<br><br>As a general rule, I would request that acronyms (e.g., "CKAN, OKFN or Git-LFS") be hyperlinked to their definitions. Otherwise it's just alphabet soup.<br><br>> Agreed, done.<br><br>Typos/grammatical errors:<br><br>p. 1 line 53: dataset on a biological organisms<br>p. 2 line 44: by adding multiple version of<br>p. 2 line 9: on a users local computer<br>p. 2 line 37: In it's present<br>p. 3 line 61: a users' analyses<br>p. 4 line 10: From the users perspective<br>p. 4 line 14: to deliver there versioned dataset<br><br>> Fixed, thank you!
**Additional Information:**	

Question	Response
Are you submitting this manuscript to a special series or article collection?	No
**Experimental design and statistics**<br><br>Full details of the experimental design and statistical methods used should be given in the Methods section, as detailed in our Minimum Standards Reporting Checklist. Information essential to interpreting the data presented should be made available in the figure legends.<br><br>Have you included all the information requested in your manuscript?	Yes
**Resources**<br><br>A description of all resources used, including antibodies, cell lines, animals and software tools, with enough information to allow them to be uniquely identified, should be included in the Methods section. Authors are strongly encouraged to cite Research Resource Identifiers (RRIDs) for antibodies, model organisms and tools, where possible.<br><br>Have you included the information requested as detailed in our Minimum Standards Reporting Checklist?	Yes
**Availability of data and materials**<br><br>All datasets and code on which the conclusions of the paper rely must be either included in your submission or deposited in publicly available repositories (where available and ethically appropriate), referencing such data using a unique identifier in the references and in the "Availability of Data and Materials" section of your manuscript.	Yes

Have you have met the above requirement as detailed in our Minimum Standards Reporting Checklist?	

Powered by Editorial Manager® and ProduXion Manager® from Aries Systems Corporation

Manuscript
Click here to access/download;Manuscript;ms.pdf
Click here to view linked References

OXFORD (GIGA)^n SCIENCE

GigaScience, 2018, 1–7

doi: xx.xxxx/xxxx
Manuscript in Preparation
Technical Note

TECHNICAL NOTE

Datastorr: a workflow and package for delivering successive versions of "evolving data" directly into R

Daniel S. Falster[1,*], Richard G. FitzJohn[2], Matthew W. Pennell[3] and William K. Cornwell[1]

[1]Evolution & Ecology Research Centre, and School of Biological, Earth and Environmental Sciences, University of New South Wales, Sydney NSW 2052, Australia and [2]Department of Infectious Disease Epidemiology, Imperial College London, Faculty of Medicine, Norfolk Place, London W2 1PG, United Kingdom and [3]Department of Zoology and Biodiversity Research Centre, University of British Columbia, Vancouver B.C. V6T 1Z4, Canada

[*]daniel.falster@unsw.edu.au

Abstract

The sharing and re-use of data has become a cornerstone of modern science. Multiple platforms now allow quick and easy data sharing. So far, however, data publishing offers limited functions for interacting with evolving datasets – those that continue to grow with time as more records are added, errors fixed, and new data structures are created. In this article, we describe a workflow for maintaining and distributing successive versions of an evolving dataset, allowing users to retrieve and load different versions directly into the R platform. Our workflow utilises tools and platforms used for development and distribution of successive versions of a open source software, including version control, GitHub and semantic versioning, and applies these to the analogous process of developing successive versions of an open source dataset. Moreover, we argue that this model allows for individual research groups to achieve a dynamic and versioned model of data delivery at no cost.

Key words: Data sharing; Version control; Semantic versioning

Introduction

Publication of a quality dataset – a collection of measurements, stored in one or several files – is now considered a first-class scientific product. Increasingly, funding bodies, publishers, and scientific social norms are recognizing the value of sharing datasets, including as standalone products without any accompanying analyses [1, 2, 3, 4, 5]. Evidence of this trend is seen in the increasing numbers of standalone "Data papers" appearing in both standard domain-level journals and specialised data journals. Yet, while the last decade has witnessed a rapid and exciting change in attitudes towards data sharing and publishing, the scientific community is still grappling with how to effectively disseminate and manage open-source datasets [1, 6, 7, 8, 9, 4, 10, 11, 12]. In particular, in some areas, such as our own area of ecology and evolution, we are only starting to support the fact that some high-quality datasets may be evolving entities [12].

An evolving (or 'living') dataset is one that is subject to recurrent or occasional change. Typical changes may include improving the quality of existing data, adding new data, re-structuring the dataset content, or integrating with other datasets. For example, a dataset on biological organisms might be expanded through the addition of new records or improved through the correction of spelling mistakes in taxonomic names. In some cases, datasets may be expected to continue to evolve over extended periods [e.g. 13]. Evolving datasets are never "finished", and as such there is no "master" or "canonical" version. Rather, as research around a data product grows, there might be many valid versions produced. Even datasets that are not initially envisioned as evolving, may become so as minor errors are identified and corrected during

Compiled on: October 10, 2018.
Draft manuscript prepared by the author.

Key Points

- Evolving datasets are those that are often being expanded and improved. Users of evolving datasets require easy ways successive versions.
- Using techniques adopted from software development, we present a workflow for maintaining and distributing versions of an evolving dataset. A new package `datastorr` enables fetching and loading successive versions directly in R.
- Using semantic versioning to label versions of dataset conveys helps users identify the type of change that has occurred.

use. In either case, the most recent version of the dataset will typically contain the best-available information, but there are many reasons to go back to previous versions: to replicate previous analyses or to work on a stable version for downstream analyses or visualisation.

A common approach taken by those maintaining an evolving dataset is to release sequential versions of the dataset, each containing a snapshot of the dataset at the time of release [e.g. 14, 15, 12, 16]. Ideally, the latest versions of an evolving dataset would be immediately available to all users across the globe, along with notes describing the changes when compared to previous versions. For the sake of reproducibility, previous versions of the dataset should be archived and remain available. In the recent past, small research groups have solved the issue of versioning data internally and informally, for example by emailing around the latest version. However, as science grows and moves towards more systematic publishing of data, scalable solutions are needed to distribute dataset versions to a wider variety of users.

One approach taken by large research consortia has been to create dedicated web servers for archiving and delivering of data. Projects such as the Sloan Digital Sky Survey (https://www.sdss.org) have sophisticated infrastructure and processes for storing and distributing successive versions of very large datasets [16]. The issue of updating and expanding published data has also been addressed in some centralised repositories, like genetic sequences (via `GenBank`), where new data can be added and there exist abilities to correct errors in existing records. Yet these web databases require a level of funding and technological infrastructure that is beyond most research groups.

The vast majority of research projects are smaller and these currently rely on more generic data repositories for publishing of data. A common approach for publishing a dataset is to release it under a Digital Object Identifier (DOI) in a standalone data repository, such as DataDryad, Figshare, and Zenodo. While these platforms did may not all initially support versioning of datasets, they now support multiple versions of a dataset, either under a single or different DOIs [17]. Yet, while these new features in principle allow users to access multiple versions of a dataset, the release, discovery, and access to multiple versions of a dataset is not always straightforward.

We believe more can be done to streamline the delivery of a potentially large number of dataset versions to users, especially for small research teams with limited budgets. There are at least three challenges. First, dataset developers need a cheap – ideally free – and reliable system to create and distribute versions of an evolving datasets with low technical overhead. Second, users need an easy mechanism to discover the existence of new (or all) versions of an evolving dataset. Third, users need a mechanism to retrieve specific versions. For those using a computational language such as R [18], all versions of an evolving dataset would ideally be both accessible and discoverable directly from within R.

In this article we outline how emerging technologies from software development (Table 1) can be used to address these challenges, enabling small research groups to create and maintain a stream of versions for small-to-medium sized datasets (up to 2Gb), and distribute these directly into the R computational environment for a potentially unlimited number of users at zero financial cost and minimal technical overhead. To achieve this we developed a new R package called `datastorr`, which together with other the technologies allows for easy and scalable delivery of successive versions of an evolving dataset directly into R. At time of publishing this article, this workflow was being used to distribute versions of datasets across a wide range of topics (Table 2), suggesting a potentially wide domain of application.

A lightweight, cheap, and scalable workflow for delivering versions of an evolving dataset into R

In brief, the workflow we present here borrows best practices for software development [19] and applies them to the challenge of maintaining and distributing versions of an evolving dataset. Our approach envisions multiple parties involved in the creation and/or use of a versioned dataset, including *developers*, *contributors* and *users* (Fig. 1). Each of these will likely have different goals and requirements (see Table 3). When building a piece of software, developers maintain a core set of code which produces the binary executable file that is eventually installed on a user's local computer. Analogously, developers of an evolving dataset maintain a core set of files (the "code"), which produces an organised dataset that can be "installed" (i.e., loaded) on a user's local computer. In either the development of software or data, successive versions – called "releases" – are distributed as snapshots of the generated product at a particular point in time.

The similarity in workflow between software and data allows us to deploy the re-purpose some of the same technological platforms that are used to maintain and distribute versions of a software product to maintain and distribute versions of an evolving dataset (Table 1). Importantly, these tools are available free of charge for open source projects and already well developed – ensuring high-level performance and stability. Moreover, the combination of technologies allow us to address the goals and requirements of the different parties involved in the creation and use of a versioned dataset (see Table 3).

An overview of the proposed system is as follows.

- Raw data files are stored under version control in a `git` repository – a free and leading version control system used in software development – by the dataset developers. All the files that go together to build a single dataset are stored in the repository, together with any code used to manipulate these files to create the dataset that is ultimately distributed.
- Changes to the raw data files and code are tracked by the developers using `git`'s ability to make "commits" – granular

Table 1. Overview of technologies used to maintain, store, and distribute versions of an evolving dataset as described in this paper.

Technology	Description
`git`	Open source version control system used for tracking progressive changes in a set of text files, typically computer code but also data.
`GitHub`	A commercial web platform available at github.com for sharing, visualising, and managing `git` repositories. Includes ability to browse the 'history', 'issue' tracking, and ability to host 'releases'. Also has a well developed Application Programming Interface (API) enabling programmatic access to dataset releases.
`R`	Widely-used and open-source language for data processing and statistical analysis.
`datastorr`	A package in `R` used to fetch releases of an evolving dataset hosted on `GitHub`.
`semantic versioning`	The process of assigning unique version numbers in a particular format to successive versions of a digital product; traditionally applied to software but here to an evolving dataset.

and annotated snapshots of the source files over time.

- The `git` repository is hosted on `GitHub` – a leading platform for hosting, enabling multiple developers or other contributors to work collaboratively on improving a dataset (Fig. 1).
- developers use the files in the repository to make a release of the dataset – a snapshot of the generated data product at a particular commit – and upload these to `GitHub`, where they are hosted alongside the raw files and (optionally) labelled using "semantic versioning". The version labels indicate both the ordering of versions and the magnitude of change expected between different versions.
- Using the `datastorr` package, users can both retrieve a list of all available versions of the dataset, and retrieve particular versions of the dataset on demand, and load them directly into `R`.

Below we elaborate on each of the different technologies.

Version control

Version control, primarily an open-source variety called `git`, has become widespread in software development. In practice, version control tracks line-by-line changes in text files and creates and maintains a history of those changes. Increasingly version control has been applied to scientific code and also data management, especially for small-to-medium sized datasets [20, 9, 8]. `git` is attractive for data management because it tracks all changes in monitored files, provided these are saved in text format (e.g., ".csv", ".tsv", ".txt"; with some tricks git can also indicate changes in some other file types such as ".xlsx"). It allows users to annotate commits with informative messages detailing the rationale for those changes. The "history" of commits is also visible to anyone interacting with the repository. In its present form, `git` can handle individual data files at least up to 100MB, which includes a large fraction of scientific cases.

As a general strategy for tracking a dataset under version control with `git`, we recommend:

- Developers establish a separate `git` repository for each dataset to be distributed.
- Saving all files as plain text, so that `git` can identify line-by-line changes. For example, save tabular data as a "csv".
- Saving data in their rawest form. In some datasets you might only have a single file. Others may have may files that get manipulated or combined in some way to produce a unified product.
- Including in the `git` repository any code needed to manipulate or compile the raw data files into the final dataset. For example, you might combine many independent datasets into one unified dataset.
- Documenting any changes in the dataset by making a commit in the `git` repository, with informative message outlin-

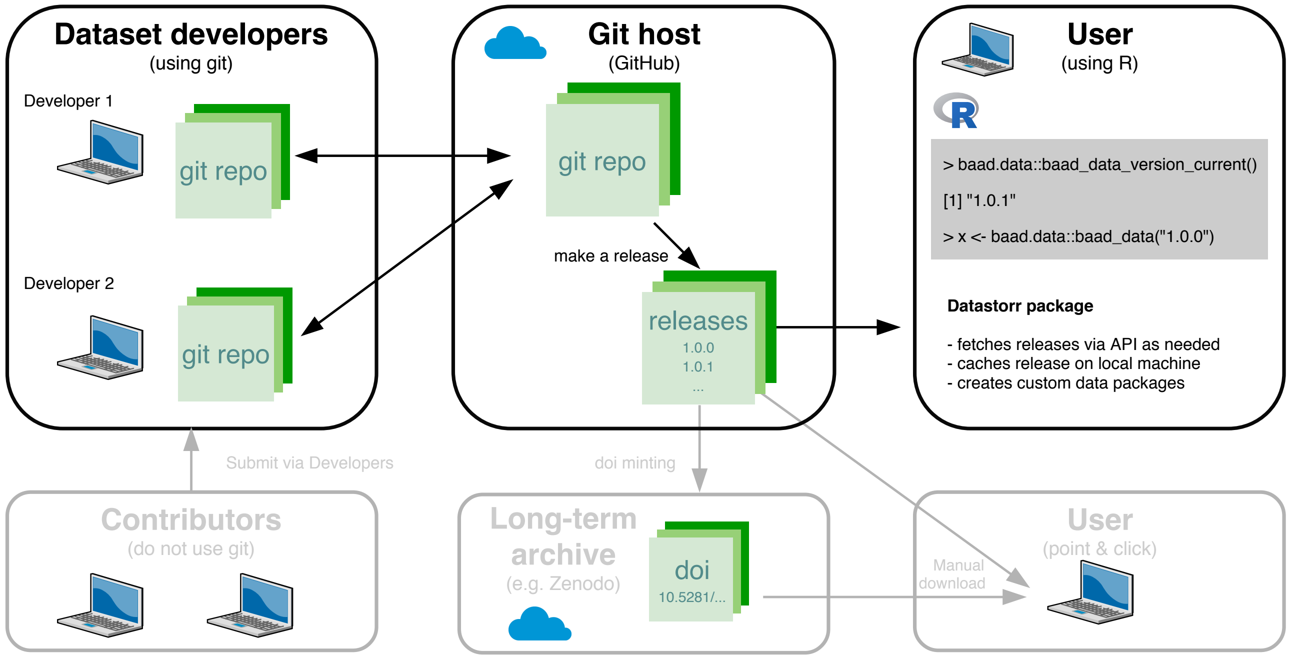

Figure 1. Overview of the workflow, different parties and technologies involved in maintaining an distributing versions of an evolving dataset via `datastorr`. Core features of our approach are shown with black boxes and arrows. Optional extensions are shown in grey (see Discussion for details).

ing why the change was made.

Hosting and distributing versions of an evolving dataset

Datasets stored under version control via `git` reach their real potential when hosted at a suitable internet hosting service [20, 9]. Here we focus on the platform `GitHub` (Table 1). Hosting of a `git` repository enables dataset developers to connect with other potential contributors and also users (Fig. 1). These platforms are designed to work with `git` repositories, and thus offer many helpful features, such as ability to record issues, host documentation, or review edits over time.

Of particular interest for current purposes, is `GitHub`'s ability to host a stream of releases from the dataset, alongside the `git` repository containing all the raw files. Each release is linked to a specific commit in the `git` repository history and occur at points where the dataset developer decided to generate a new version of the data for distribution. While users could in principle download the entire `git` repository, actually all they want (most of the time) are the releases.

Deciding when to make a new release is at the discretion of the dataset developer. In practice, one makes fewer releases than one does commits into the `git` repository, though there is nothing stopping developers from releasing a new version for every commit. The flexibility here allows developers to do internal work between releases and only release the data to users when the revision represents a clear improvement on the previous release.

Another important consideration is that websites like `GitHub` naturally cater for two types of data users accessing the data: those that interact with the data via point and click downloading, and those that use programmatic interaction (Fig. 1, Table 3). Specifically, `GitHub` releases can be downloaded directly by users or accessed programmatically via the `GitHub` API.

Semantic versioning

To realize the full benefits of a versioned controlled dataset, users should be able to easily intuit the types of changes that have occurred among versions. Since software development has effectively already dealt with a similar problem in the labelling of software releases, we suggest there is benefit in adopting the best-practices from that field.

Specifically, we suggest adapting the process of semantic versioning, developed for software distribution (semver.org), to labelling of successive releases of an evolving dataset (Fig 2). Semantic versioning uses a tri-digit notation of the form "X.Y.Z" for successive versions, where X, Y, and Z are non-negative integers. For example, version "2.1.2". Software developers increment particular numbers in a systematic way to communicate any changes to the public API for a particular piece of software.

There are two natural advantages of semantic versioning of adapting the process of semantic versioning for dataset development. The first is that enables natural ordering of releases.

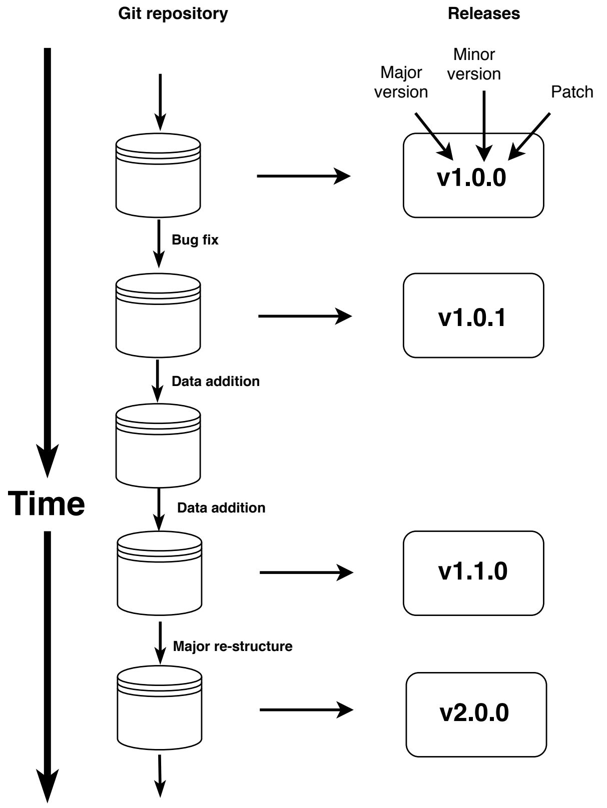

Figure 2. Semantic versioning allows dataset developers to communicate to users the types of changes that have occurred between successive versions of an evolving dataset.

The second, is that it enables developers to signal the type and magnitude of change that occurred in the product between successive versions. Seeing a series of version numbers, users of an evolving dataset know the developer's view on the type and/or magnitude of change between versions.

Though the analogy to software is not perfect, datasets can also be thought of as having an Interface, which is the structure that allows for interacting with the actual data. For example, in tabular data the structure would be the column and row labels, and the data the actual cell contents. Changes to the Interface an then be labelled with semantic versioning.

Building off the guidelines for semantic versioning of software, we suggest the following guidelines for labelling a dataset with semantic versioning.

- Clearly communicate the Interface to the dataset in the metadata or landing page. This includes file types, dataset structure, and element names.
- Use versions beginning with "0.Y.Z" to indicate products where the Interface is still in development.
- Version "1.0.0" defines the public Interface.

Table 2. Example datasets currently delivered using the `datastorr` package for `R`

`GitHub` **repository**	**Dataset description**
dfalster/baad.data	Size dimensions of plants for many species from across the world [14].
traitecoevo/taxonlookup	Taxonomy of world's land plants [15].
ecohealthalliance/cites	Trade details from Convention on International Trade in Endangered Species (CITES)
madams1/nbadata	Statistics from the National Basketball Association (NBA) seasons 1996-97 to 2016-17
madams1/floridainmates	Dataset from the Florida Department of Corrections on the state's inmate population
traitecoevo/fungaltraits	Dataset on the traits of fungi [21].

- Once defined, increment version numbers to communicate any changes to the Interface.
- Increment the "Major" version when you make changes to the Interface that are likely incompatible with any code written to work with previous versions (i.e. causes an error). Such changes may include revising the file names, the structure of the dataset, or changing element names (e.g. column headers).
- Increment the "Minor" version to communicate any changes to the Interface that are likely to be compatible with any code written to work with the previous versions (i.e. allows code to run without error). Such changes typically involve adding new data within the existing structure, so that the previous dataset version exists as a subset of the new version. For tabular data, this includes adding columns or rows. On the other hand, removing data should constitute a major version, as records previously relied on may no longer exists.
- Increment the "Patch" version to communicate correction of errors in the actual data, without any changes to the Interface. Such changes are unlikely to break, or change in a substantial way, analyses written with the previous version, although there may of course be changes in the results.
- Once a dataset version has been released, do not modify it. Further modifications are released under a new version number.

While these guidelines will help users in understanding the types of changes that have occurred between successive versions of a dataset, *any* change in a dataset may alter the results of a users' analysis in non-trivial ways. Unlike developers of software, developers of a dataset cannot guarantee full backwards-compatibility, i.e. that certain results will remain unchanged in updated versions. We suggest responsibility for verifying how different versions of an evolving dataset influence their particular analysis or use thus always remains with the user, even if simply applying a so-called "patch". While further work – and likely experience – is needed to refine the process of semantic versioning for datasets to further develop understanding between data developers and data users of what different changes imply, semantic versioning still provides a more nuanced way to communicate from the developer to the user on the types of change they could expect.

Loading data versions directly into `R` using the `datastorr` package

For efficient usage and to aid reproducibility, many users will want access to all versions of any particular dataset programmatically (Table 3). Code to access a stream of `GitHub` releases could be written individually by each user, but this creates an unnecessary technological hurdle. To make it easier for users to access versioned data via code, we developed a new package for the `R` platform, as one of the most prominent platforms for data science [18].

Our package, called `datastorr` (github.com/ropenscilabs/datastorr), facilitates access to releases of any evolving dataset hosted on `GitHub` (Fig. 1). Specifically, the `datastorr` package: 1) Contains the main code needed to interact with the `GitHub` API to retrieve versions of the dataset; and 2) Enables users to construct the shell of a second, dataset-specific `R` package, which can be distributed and used to access releases for a specific repository stored on `GitHub`. Using `datastorr`, a researcher can create and distribute a custom `R` package that facilitates access to their data with (very) minimal computational skills.

For example, `datastorr` has been used to build several packages (Table 2), including `baad.data` (github.com/traitecoevo/baad.data), which is an interface to the Biomass and Allometry Database [14] stored at github.com/dfalster/baad. The R package `baad.data` consists of only a few simple functions and associated help files, that were automatically generated with `datastorr`. For a user, accessing a version of the data is a simple as typing a single line of code (Fig. 1). Accessing a different version of the data involves changing only the version number. From the user's perspective, the existence of the `baad.data` and `datastorr` packages makes reproducing analyses using specific versions of the data possible [e.g.][][22, 23].

Using `datastorr`, dataset developers can set up their own `R` package to deliver versions of an evolving dataset simply by providing:

i. a `GitHub` repository name (e.g., "traitecoevo/baad.data") where releases are stored;
ii. the filename in the release that contains data;
iii. the function used to load the data file into `R`.

Then as the dataset grows over time, the developers update the `git` repository and create a `GitHub` release with a new version number. All the releases are simultaneously available to any user, both point-and-click and programmatically.

The dataset-specific packages created by `datastorr` are designed to be computationally efficient and also work offline. Packages created by `datastorr` contain no actual data, only the rules for fetching the data. As such, the basic package structure is quick to install and takes up virtually no space on the user's hard-drive. The package functions by fetching each data version once (the first time it is requested), and then caching these files locally for future reuse. Moreover, users can store several versions of an evolving dataset on their computer and unambiguously access different versions with single function.

Discussion

The key issue we are dealing with in this article may be familiar to many readers: many datasets are constantly evolving and, despite tremendous advances in data sharing and associated technologies over the last decade, there is as yet little consensus about how to maintain and distribute multiple versions of an evolving dataset, especially for small research teams. While such teams could in principle create their own dynamic web interface, the technological hurdles, cost and maintenance required are discouraging. Moreover, existing data sharing platforms offer a limited set of features for the delivery of successive versions of an evolving dataset. This suggests there is a substantial need for an easy, cheap, and scalable solution for maintaining and distributing successive versions of an evolving dataset. By adopting open-source and scalable practices from software development, we believe a workable system already largely exists. To aid this process, we created the `datastorr` package to deliver dataset versions directly into the `R` environment. The approach and package is already being used to deliver versions of several evolving datasets spanning a wide range of topics (Table 2). Moreover, as it builds off established and open source software and data science platforms (Table 1), the proposed system is already easy to deploy on relatively large scale.

Towards an ecosystem for evolving data

Our contribution here connects with a growing number of recommendations and technologies supporting the publishing and reuse of evolving data. Such contributions include community

Table 3. Goals and requirements of different parties involved in creating and using and evolving dataset.

Group	Primary goal	Requirements
Developer	Create and distribute versions of an evolving dataset	Low technical overhead
		Low initial and ongoing cost and maintenance
		Easy workflow for releasing new versions
		Enable user feedback in error checking and contributions
		Long term preservation
Contributor	Contribute to future versions of an evolving dataset	Add new data
		Report errors in existing data
Users (all)	Easy access to all versions of an evolving dataset	Access metadata and background information
		Access to all versions of a dataset
		Ability to give feedback and contribute
		Long term stability
Users (programmatic)	As above, plus	
		Programmatic access to all versions of an evolving dataset
		Reproduce products using specific versions of an evolving dataset
		Easy installation

guidance on good practice in data curation [6, 8], data citation [7] and the FAIR principles for making datasets Findable, Accessible, Interoperable, and Reusable by both machines and humans [11]. In our proposed system, information about appropriate attribution for any dataset (whatever that is determined to be) should be made readily available, either on the landing page within GitHub, or even better distributed as part of the versioned dataset itself. Similarly, datasets can be structured to make them follow the FAIR principles, to the extent possible. Notably, our workflow with the `datastorr` package demands machine access to datasets – a core focus for the FAIR principles. While our proposed workflow does not currently enhance discoverability of new datasets, this is a broad challenge faced by all data platforms and researchers.

While our package `datastorr` offers an easy way for users of the `R` ecosystem to directly access dataset version, users of other languages can also access the datasets. Moreover, packages similar to `datastorr` would ideally be developed to make accessing dataset versions as easy as it is with `datastorr`.

Within the `R` ecosystem, the `datastorr` package complements other approaches for creating and delivering datasets. One common approach used within `R` is to embed data directly within an `R` package, which can then be distributed via the Comprehensive R Archive Network (cran.r-project.org). Moreover, dedicated packages are being developed to assist dataset developers in creating data packages [24]. An advantage of this approach, compared to ours, is that the data are immediately available in the package (whereas our packages only contain instructions for fetching the data). This advantage however also brings limitations. Notably, datasets must be under 5MB, and only one version of a dataset package can be installed on any given machine at any one time. `datastorr` offers a viable approach for overcoming these limitations.

There are also many emerging or alternative technologies that offer other possible ways to implement a system for storing and distributing versions of an evolving dataset. Our solution currently emphasises the platform GitHub, but similar functions could be achieved via other `git` hosts such as bitbucket.org and gitlab.com. Git repositories can also be extended to accommodate larger files using features like Git-Large File Storage or git-annex. More fundamentally, there are emerging alternatives for version control specifically designed for data, such as the datproject.org, and other new platforms for distributing data, such as the Comprehensive Knowledge Archive Network, (CKAN) and Open Knowledge International, (OKFN).

The key here is not the specific technology, but rather the concept of creating, maintaining, and distributing versions of an evolving dataset, which may be achieved with all of these approaches. Indeed, as with every technology the best available approach is certain to evolve, especially as emerging technologies facilitate even better delivery of data in the future.

Further advantages and extensions

A central feature of the proposed system is that data are maintained in the cloud. This has two main benefits: first, it provides a platform for multiple data contributors to sync their files and correspond about changes in the dataset, and second, it allows for hosting of a stream of data releases for distribution (Fig. 1). Cloud systems thus act as a central point for the collection, curation, and distribution of the data. Additionally, one of the greatest benefits of using cloud-based tools like `GitHub` for development of software and data has been the way they encourage contributions from multiple individuals working simultaneously — including from people from outside the initial group of project participants [25, 9]. Multiple developers can make changes to different parts of the code (or, in our case, data) and the `git` system will integrate these together or, when needed, flag where there are conflicts that need to be resolved. The proposed system of data delivery thus has the added benefit of facilitating seamless and transparent collaboration among research groups in the construction and maintenance of datasets.

An important concern for any data delivery system is the stability and reliability of the system. In the short term, users want minimal downtime, high speed, and seamless operation. As one of the largest companies hosting computer source code, `GitHub` provides exceptional performance in this regard – certainly as good or better than nearly any system scientists might build themselves. Thus, short-term concerns of reliable and fast performance are almost guaranteed.

In the long term, scientists want their datasets, software, and papers to preserved and remain accessible. While our proposed system for data delivery does not guarantee long-term preservation, users can also choose to automatically archive data-versions released on `GitHub` version in one of several traditional data archives, with a DOI (Digital Object Identifier) minted for each release. Currently, both `Zenodo` and `FigShare` each integrate with `GitHub` for archiving of material hosted there. Ideally, tools like `datastorr` would also be developed to pull versions from these archives too.

Availability of source code and requirements

- Project name: `datastorr`
- Project home page: github.com/ropenscilabs/datastorr.
- Operating system(s): Platform independent
- Programming language: R
- License: MIT

Declarations

List of abbreviations

API = An Application Programming Interface provides a set of protocols for exchanging information. DOI = Digital Object Identifier.

Ethical Approval

Not applicable.

Consent for publication

Not applicable.

Competing Interests

The author(s) declare that they have no competing interests.

Funding

DSF was funded by the Australian Research Council. MWP was funded by a NSERC Discovery Grant.

Author's Contributions

RF developed the datastorr package. All authors discussed the concepts and wrote the paper.

Acknowledgements

We thank D Noble for comments on an earlier draft and C Boettiger for helpful discussions.

References

1. Whitlock MC. Data archiving in ecology and evolution: best practices. Trends in Ecology & Evolution 2011;26:61–65.
2. Fairbairn DJ. The advent of mandatory data archiving. Evolution 2011;65:1–2.
3. Piwowar HA, Vision TJ, Whitlock MC. Data archiving is a good investment. Nature 2011;473:285–285.
4. Van Noorden R. Data-sharing: everything on display. Nature 2013;500:243–245.
5. Gibney E, Van Noorden R, Scientists losing data at a rapid rate; 2013. Nature news. doi: 10.1038/nature.2013.14416.
6. Goodman A, Pepe A, Blocker AW, Borgman CL, Cranmer K, Crosas M, et al. Ten simple rules for the care and feeding of scientific data. PLoS Comput Biol 2014;10:e1003542.
7. Data Citation Synthesis Group, Martone M. Joint declaration of data citation principles. Force11; 2014.
8. Lowndes JSS, Best BD, Scarborough C, Afflerbach JC, Frazier MR, O'Hara CC, et al. Our path to better science in less time using open data science tools. Nature Ecology & Evolution 2017;1:0160.
9. Perkel J. Democratic databases: science on GitHub. Nature 2016;538:127–128.
10. Kratz JE, Strasser C. Making data count. Scientific Data 2015;2:sdata201539.
11. Wilkinson MD, Dumontier M, Aalbersberg IJ, Appleton G, Axton M, Baak A, et al. The FAIR guiding principles for scientific data management and stewardship. Scientific Data 2016;3:sdata201618.
12. Yenni GM, Christensen EM, Bledsoe EK, Supp SR, Diaz RM, White EP, et al. Developing a modern data workflow for evolving data. bioRxiv 2018 Jul;p. 344804.
13. Ernest SM, Yenni GM, Allington G, Bledsoe E, Christensen E, Diaz R, et al. The Portal Project: a long-term study of a Chihuahuan desert ecosystem. bioRxiv 2018;p. 332783.
14. Falster DS, Duursma RA, Ishihara MI, Barneche DR, FitzJohn RG, Vårhammar A, et al. BAAD: a Biomass And Allometry Database for woody plants. Ecology 2015;96:1445.
15. Pennell MW, FitzJohn RG, Cornwell WK. A simple approach for maximizing the overlap of phylogenetic and comparative data. Methods in Ecology and Evolution 2015;7:751–758.
16. Abolfathi B, Aguado DS, Aguilar G, Prieto CA, Almeida A, Ananna TT, et al. The fourteenth data release of the Sloan Digital Sky Survey: first spectroscopic data from the extended Baryon oscillation spectroscopic survey and from the second phase of the Apache point observatory galactic evolution experiment. The Astrophysical Journal Supplement Series 2018;235(2):42.
17. Nielsen LH, Zenodo now supports DOI versioning!; 2017. OpenAIRE blog. https://blogs.openaire.eu/?p=2010.
18. R Core Team. R: a language and environment for statistical computing. R Foundation for Statistical Computing, Vienna, Austria; 2017, http://www.R-project.org/.
19. Perez-Riverol Y, Gatto L, Wang R, Sachsenberg T, Uszkoreit J, da Veiga Leprevost F, et al. Ten simple rules for taking advantage of git and gitHub. PLOS Comput Biol 2016;12:e1004947.
20. Ram K. Git Can Facilitate Greater Reproducibility and Increased Transparency in Science. Source Code for Biology and Medicine 2013;8:7.
21. Cornwell WK, Flores-Moreno H, traitecoevo/fungaltraits v0.0.3; 2018. https://doi.org/10.5281/zenodo.1216257.
22. Duursma RA, Falster DS. Leaf mass per area, not total leaf area, drives differences in above-ground biomass distribution among woody plant functional types. New Phytologist 2016;212:368–376.
23. Falster DS, Duursma RA, FitzJohn RG. How functional traits influence plant growth and shade tolerance across the life cycle. Proceedings of the National Academy of Sciences 2018;p. 201714044.
24. Finak G, Mayer B, Fulp W, Obrecht P, Sato A, Chung E, et al. DataPackageR: Reproducible data preprocessing, standardization and sharing using R/Bioconductor for collaborative data analysis. Gates Open Research 2018;2:31.
25. Rogers M, The Github revolution: why we're all in open source now; 2013. https://www.wired.com/2013/03/github/.

Figure 1

Click here to access/download;Figure;Figure-stack.pdf

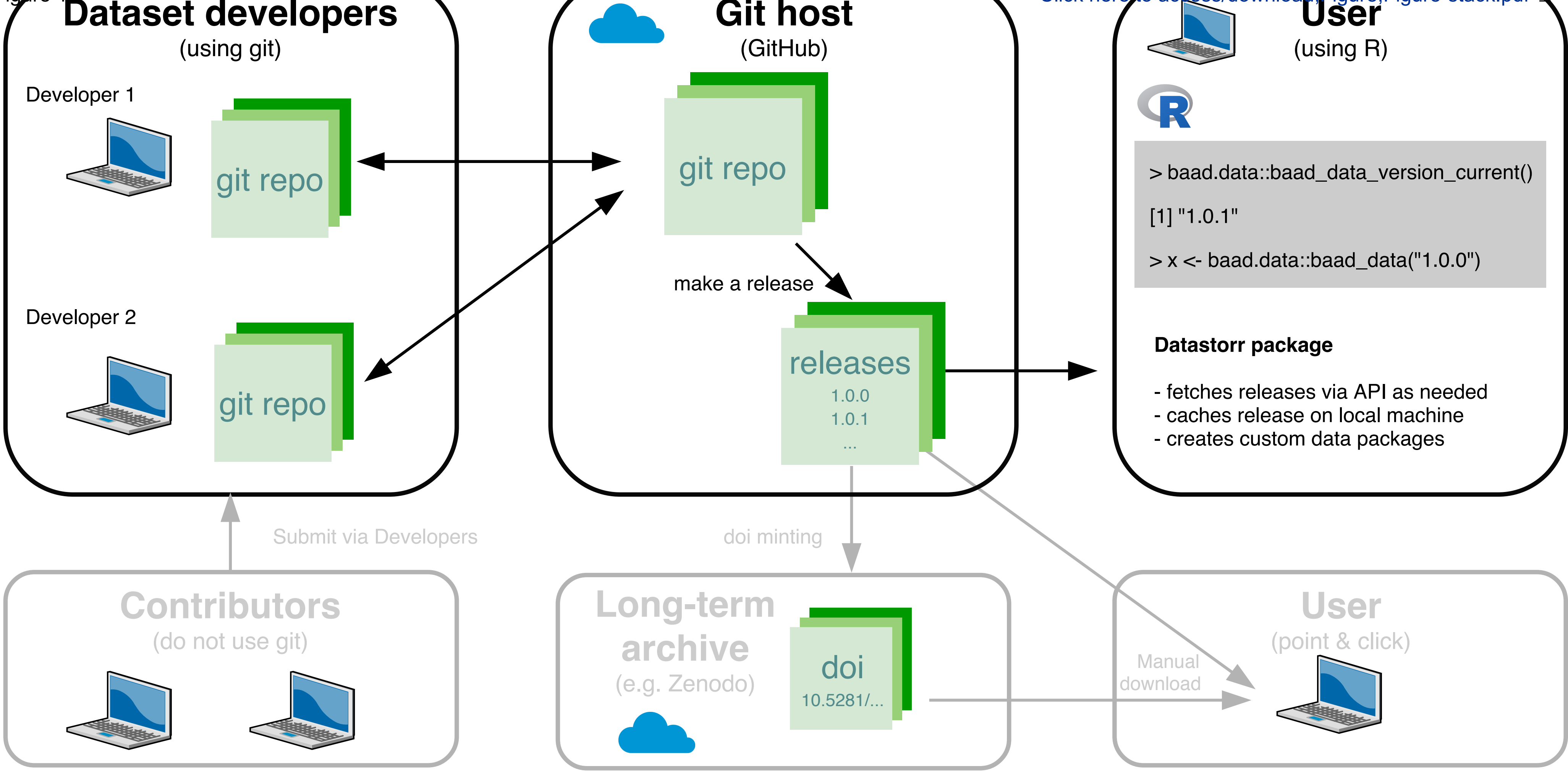

Figure 2

Click here to access/download;Figure;Figure

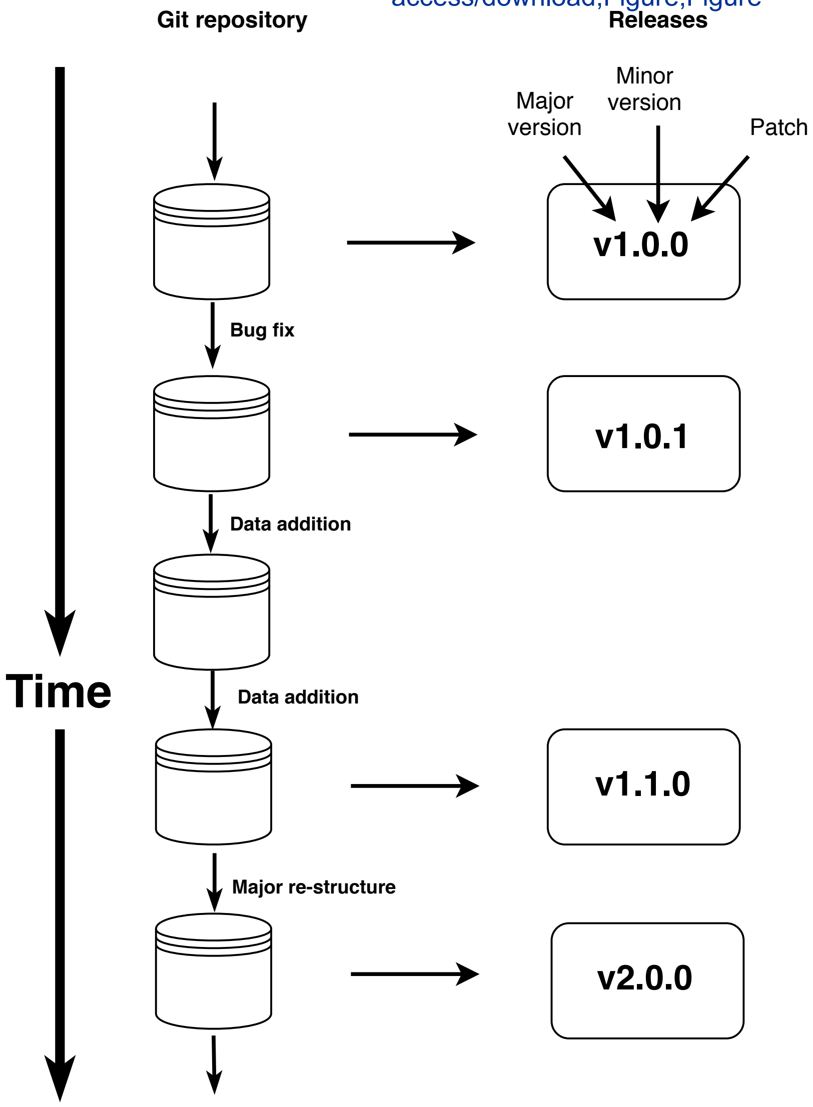

Supplement: GIGA-D-18-00005_Revision_1.pdf [file giz035_giga-d-18-00005_revision_1.pdf]
