# Supplementary material for: Datastorr: a workflow and package for delivering successive versions of 'evolving data' directly into R
Source: Gigascience. 2019 May 1;8(5):giz035. doi: 10.1093/gigascience/giz035 (PMC6506717; doi:10.1093/gigascience/giz035)

GigaScience

Datastorr: a workflow and package for delivering successive versions of 'evolving data' directly into R

--Manuscript Draft--

**Manuscript Number:**	GIGA-D-18-00005R3
**Full Title:**	Datastorr: a workflow and package for delivering successive versions of 'evolving data' directly into R
**Article Type:**	Technical Note
**Funding Information:**	
**Abstract:**	The sharing and re-use of data has become a cornerstone of modern science. Multiple platforms now allow quick and easy data sharing. So far, however, data publishing offers limited functions for interacting with evolving datasets - those that continue to grow with time as more records are added, errors fixed, and new data structures are created. In this article, we describe a workflow for maintaining and distributing successive versions of an evolving dataset, allowing users to retrieve and load different versions directly into the R platform. Our workflow utilises tools and platforms used for development and distribution of successive versions of a open source software, including version control, GitHub and semantic versioning, and applies these to the analogous process of developing successive versions of an open source dataset. Moreover, we argue that this model allows for individual research groups to achieve a dynamic and versioned model of data delivery at no cost.
**Corresponding Author:**	Daniel S. Falster, PhD<br><br>AUSTRALIA
**Corresponding Author Secondary Information:**	
**Corresponding Author's Institution:**	
**Corresponding Author's Secondary Institution:**	
**First Author:**	Daniel Falster
**First Author Secondary Information:**	
**Order of Authors:**	Daniel Falster
	Richard FitzJohn
	Matthew Pennell
	William Cornwell
**Order of Authors Secondary Information:**	
**Response to Reviewers:**	Minor changes as requested, including<br><br>- add phone for correpsonding author<br>- add ORCID ids<br>- add link to supporting data package<br>- add details for two other supporting software packages included in supporting data package<br>- add extra items to List of abbreviations<br>- add grant numbers<br>- references: add URLS and update paper now published. Reference data package
**Additional Information:**	
**Question**	**Response**
Are you submitting this manuscript to a	No

special series or article collection?	
**Experimental design and statistics**<br><br>Full details of the experimental design and statistical methods used should be given in the Methods section, as detailed in our Minimum Standards Reporting Checklist. Information essential to interpreting the data presented should be made available in the figure legends.<br><br>Have you included all the information requested in your manuscript?	Yes
**Resources**<br><br>A description of all resources used, including antibodies, cell lines, animals and software tools, with enough information to allow them to be uniquely identified, should be included in the Methods section. Authors are strongly encouraged to cite Research Resource Identifiers (RRIDs) for antibodies, model organisms and tools, where possible.<br><br>Have you included the information requested as detailed in our Minimum Standards Reporting Checklist?	Yes
**Availability of data and materials**<br><br>All datasets and code on which the conclusions of the paper rely must be either included in your submission or deposited in publicly available repositories (where available and ethically appropriate), referencing such data using a unique identifier in the references and in the "Availability of Data and Materials" section of your manuscript.<br><br>Have you have met the above requirement as detailed in our Minimum Standards Reporting Checklist?	Yes

Powered by Editorial Manager® and ProduXion Manager® from Aries Systems Corporation

Manuscript
Click here to access/download;Manuscript;ms.pdf
Click here to view linked References

GigaScience, 2018, 1–8

doi: xx.xxxx/xxxx
Manuscript in Preparation
Technical Note

TECHNICAL NOTE

Datastorr: a workflow and package for delivering successive versions of "evolving data" directly into R

Daniel S. Falster[1,*], Richard G. FitzJohn[2], Matthew W. Pennell[3] and William K. Cornwell[1]

[1]Evolution & Ecology Research Centre, and School of Biological, Earth and Environmental Sciences, University of New South Wales, Sydney NSW 2052, Australia and [2]Department of Infectious Disease Epidemiology, Imperial College London, Faculty of Medicine, Norfolk Place, London W2 1PG, United Kingdom and [3]Department of Zoology and Biodiversity Research Centre, University of British Columbia, Vancouver B.C. V6T 1Z4, Canada

*daniel.falster@unsw.edu.au, +61-2-93858431
ORCID IDs. Daniel S Falster: 0000-0002-9814-092X, Matthew W. Pennell: 0000-0002-2886-3970, William K. Cornwell: 0000-0003-4080-4073

Abstract

The sharing and re-use of data has become a cornerstone of modern science. Multiple platforms now allow easy publication of datasets. So far, however, platforms for data sharing offers limited functions for distributing and interacting with evolving datasets – those that continue to grow with time as more records are added, errors fixed, and new data structures are created. In this article, we describe a workflow for maintaining and distributing successive versions of an evolving dataset, allowing users to retrieve and load different versions directly into the R platform. Our workflow utilises tools and platforms used for development and distribution of successive versions of a open source software, including version control, GitHub and semantic versioning, and applies these to the analogous process of developing successive versions of an open source dataset. Moreover, we argue that this model allows for individual research groups to achieve a dynamic and versioned model of data delivery at no cost.

Key words: Data sharing; Version control; Semantic versioning

Introduction

Sharing of a quality dataset – a collection of measurements, stored in one or several files – is now considered a first-class scientific output. Increasingly, funding bodies, publishers, and scientific social norms are recognizing the value of sharing datasets, including as standalone products without any accompanying analyses [1, 2, 3, 4, 5]. Evidence of this trend is seen in the increasing numbers of standalone "Data papers" appearing in both standard domain-level journals and specialised data journals. Yet, while the last decade has witnessed a rapid and exciting change in attitudes towards data sharing, the scientific community is still grappling with how to effectively maintain and distribute open-source datasets [1, 6, 7, 8, 9, 4, 10, 11, 12]. In particular, in some areas, such as our own area of ecology and evolution, we are only starting to support the fact that some high-quality datasets may be evolving entities [12].

An evolving (or 'living') dataset is one that is subject to occasional or recurrent change. Typical changes may include improving the quality of existing data, adding new data, re-structuring the dataset content, or integrating with other datasets. For example, a dataset on biological organisms might be expanded through the addition of new records or improved through the correction of spelling mistakes in taxonomic names. In some cases, datasets may be expected to continue to evolve over extended periods [e.g. 13]. Evolving datasets are never "finished", and as such there is no "master" or "canonical" version. Rather, as research around a data

Compiled on: March 13, 2019.
Draft manuscript prepared by the author.

Key Points

- Evolving datasets are those that are often being expanded and improved. Users of evolving datasets require easy ways successive versions.
- Using techniques adopted from software development, we present a workflow for maintaining and distributing versions of an evolving dataset. A new package `datastorr` enables fetching and loading successive versions directly in R.
- Using semantic versioning to label versions of dataset conveys helps users identify the type of change that has occurred.

product grows, there might be many valid versions produced. Even datasets that are not initially envisioned as evolving, may become so as minor errors are identified and corrected during use. In either case, the most recent version of the dataset will typically contain the best-available information, but there are still reasons to go back to previous versions: to replicate previous analyses or to work on a stable version for downstream analyses or visualisation.

A common approach taken by those maintaining an evolving dataset is to release sequential versions of the dataset, each containing a snapshot of the dataset at the time of release [e.g. 14, 15, 12, 16]. Ideally, the latest versions of an evolving dataset would be immediately available to all users across the globe, along with notes describing the changes when compared to previous versions. For the sake of reproducibility, previous versions of the dataset should be archived and remain available. In the recent past, small research groups have solved the issue of versioning data internally and informally, for example by emailing around the latest version. However, as science grows and moves towards more systematic sharing of data, scalable solutions are needed to distribute dataset versions to a wider variety of users.

One approach taken by large research consortia has been to create dedicated web servers for archiving and delivering of data. Projects such as the Sloan Digital Sky Survey (https://www.sdss.org) have sophisticated infrastructure and processes for distributing successive versions of very large datasets [16]. The issue of updating data has also been addressed in some centralised repositories, like genetic sequences (via `GenBank`), where new data can be added and there exist abilities to correct errors in existing records. Yet these web databases require a level of funding and technological infrastructure that is beyond most research groups.

The vast majority of research projects are smaller and these currently rely on more generic data repositories for distributing data. A common approach for distributing a dataset is to release it under a Digital Object Identifier (DOI) in a standalone data repository, such as DataDryad, Figshare, and Zenodo. While these platforms did not all initially support versioning of datasets, they now support multiple versions of a dataset, either under a single or different DOIs [17]. Yet, while these new features in principle allow users to access multiple versions of a dataset, the release, discovery, and access to multiple versions of a dataset is not always straightforward.

We believe more can be done to streamline the distribution of a potentially large number of dataset versions to users, especially for small research teams with limited budgets. There are at least three challenges. First, dataset developers need a cheap – ideally free – and reliable system to create and distribute versions of an evolving datasets with low technical overhead. Second, users need an easy mechanism to discover the existence of new (or all) versions of an evolving dataset. Third, users need a mechanism to retrieve specific versions. For those using a computational language such as R [18], all versions of an evolving dataset would ideally be both accessible and discoverable directly from within R.

In this article we outline how emerging technologies from software development (Table 1) can be used to address these challenges, enabling small research groups to create and maintain a stream of versions for small-to-medium sized datasets (up to 2Gb), and distribute these directly into the R computational environment for a potentially unlimited number of users at zero financial cost and minimal technical overhead. To achieve this we developed a new R package called `datastorr`, which together with other the technologies allows for easy and scalable delivery of successive versions of an evolving dataset directly into R. At time of publishing this article, this workflow was being used to distribute versions of datasets across a wide range of topics (Table 2), suggesting a potentially wide domain of application.

A lightweight, cheap, and scalable workflow for delivering versions of an evolving dataset into R

In brief, the workflow we present here borrows best practices for software development [19] and applies them to the challenge of maintaining and distributing versions of an evolving dataset. Our approach envisions multiple parties involved in the creation and/or use of a versioned dataset, including *developers*, *contributors* and *users* (Fig. 1). Each of these will likely have different goals and requirements (see Table 3). When building a piece of software, developers maintain a core set of code which produces the binary executable file that is eventually installed on a user's local computer. Analogously, developers of an evolving dataset maintain a core set of files (the "code"), which produces an organised dataset that can be "installed" (i.e., loaded) on a user's local computer. In the development of either software or data, successive versions – called "releases" – are distributed as snapshots of the generated product at a particular point in time.

The similarity in workflow between software and data allows us to deploy the re-purpose some of the same technological platforms that are used to maintain and distribute versions of a software product to maintain and distribute versions of an evolving dataset (Table 1). Importantly, these tools are available free of charge for open source projects and already well developed – ensuring high-level performance and stability. Moreover, the combination of technologies allow us to address the goals and requirements of the different parties involved in the creation and use of a versioned dataset (see Table 3).

An overview of the proposed system is as follows.

- Raw data files are stored under version control in a `git` repository – a free and leading version control system used in software development – by the dataset developers. All the files that go together to build a single dataset are stored in the repository, together with any code used to manipulate these files to create the dataset that is ultimately distributed.

Table 1. Overview of technologies used to maintain, store, and distribute versions of an evolving dataset as described in this paper.

Technology	Description
`git`	Open source version control system used for tracking progressive changes in a set of text files, typically computer code but also data.
`GitHub`	A commercial web platform available at github.com for sharing, visualising, and managing `git` repositories. Includes ability to browse the 'history', 'issue' tracking, and ability to host 'releases'. Also has a well developed Application Programming Interface (API) enabling programmatic access to dataset releases.
`R`	Widely-used and open-source language for data processing and statistical analysis.
`datastorr`	A package in `R` used to fetch releases of an evolving dataset hosted on `GitHub`.
`semantic versioning`	The process of assigning unique version numbers in a particular format to successive versions of a digital product; traditionally applied to software but here to an evolving dataset.

- Changes to the raw data files and code are tracked by the developers using `git`'s ability to make "commits" – granular and annotated snapshots of the source files over time.
- The `git` repository is hosted on `GitHub` – a leading platform for hosting, enabling multiple developers or other contributors to work collaboratively on improving a dataset (Fig. 1).
- Developers use the files in the repository to make a release of the dataset – a snapshot of the generated data product at a particular commit – and upload these to `GitHub`, where they are hosted alongside the raw files and (optionally) labelled using "semantic versioning". The version labels indicate both the ordering of versions and the magnitude of change expected between different versions.
- Using the `datastorr` package, users can both retrieve a list of all available versions of the dataset, and retrieve particular versions of the dataset on demand, and load them directly into `R`.
- Those not using `R` can also access versions from `GitHub`.

Below we elaborate on each of the different technologies.

Version control

Version control, primarily an open-source variety called `git`, has become widespread in software development. In practice, version control tracks line-by-line changes in text files and creates and maintains a history of those changes. Increasingly version control has been applied to scientific code and also data management, especially for small-to-medium sized datasets [20, 9, 8]. `git` is attractive for data management because it tracks all changes in monitored files, provided these are saved in text format (e.g., ".csv", ".tsv", ".txt"; with some tricks git can also indicate changes in some other file types such as ".xlsx"). It allows users to annotate commits with informative messages detailing the rationale for those changes. The "history" of commits is also visible to anyone interacting with the repository. In its present form, `git` can handle individual data files at least up to 100MB, which includes a large fraction of scientific cases.

As a general strategy for tracking a dataset under version control with `git`, we recommend:

- Developers establish a separate `git` repository for each dataset to be distributed.
- Saving data in their rawest form. In some datasets you might only have a single file. Others may have may files that get manipulated or combined in some way to produce a unified product.
- Where possible, saving all files as plain text, so that `git` can identify line-by-line changes. For example, save tabular data as a "csv". While this approach works well for small-to-intermediate sized files, those with larger files may prefer to use a compressed format to reduce repository size and

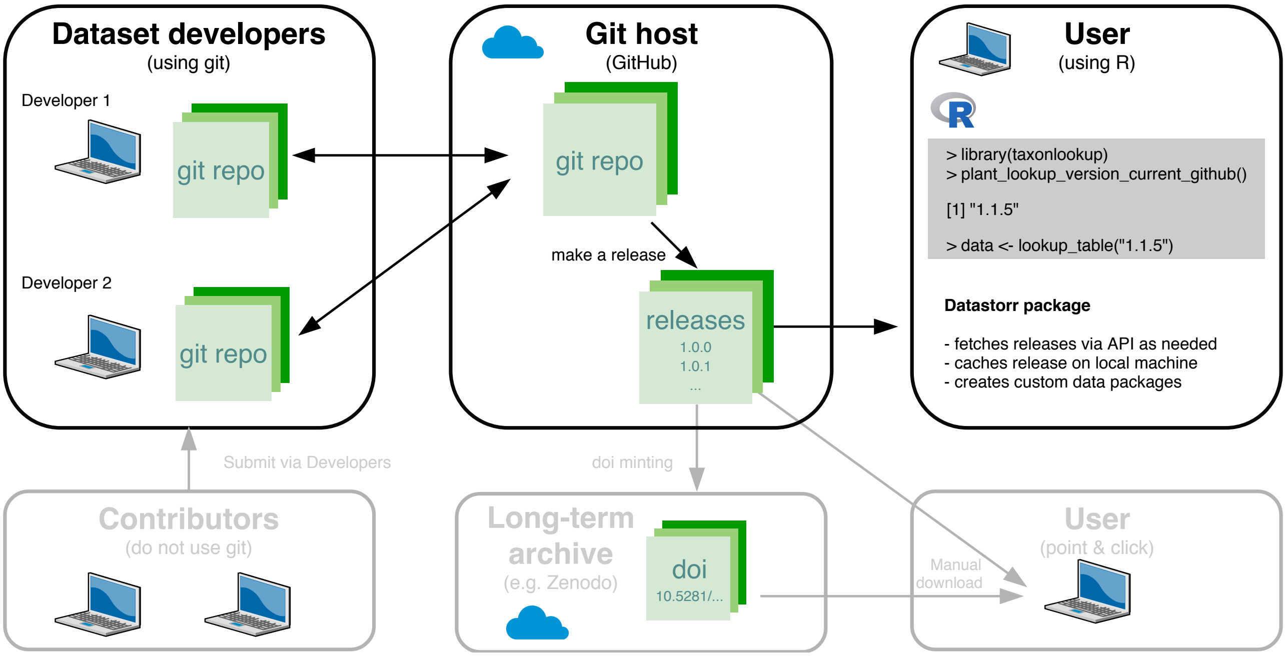

Figure 1. Overview of the workflow, different parties and technologies involved in maintaining an distributing versions of an evolving dataset via `datastorr`. Core features of our approach are shown with black boxes and arrows. Optional extensions are shown in grey (see Discussion for details).

bandwidth.

- Including in the `git` repository any code needed to manipulate or compile the raw data files into the final dataset. For example, you might combine many independent datasets into one unified dataset.
- Documenting any changes in the dataset by making a commit in the `git` repository, with informative message outlining why the change was made.

Hosting and distributing versions of an evolving dataset

Datasets stored under version control via `git` reach their real potential when hosted at a suitable internet hosting service [20, 9]. Here we focus on the platform `GitHub` (Table 1). Hosting of a `git` repository enables dataset developers to connect with other potential contributors and also users (Fig. 1). These platforms are designed to work with `git` repositories, and thus offer many helpful features, such as ability to record issues, host documentation, or review edits over time.

Another notable feature of `GitHub` is the ability to host a stream of releases from the dataset, alongside the `git` repository containing all the raw files. Each release is linked to a specific commit in the `git` repository history and occur at points where the dataset developer decided to generate a new version of the data for distribution. While users could in principle download the entire `git` repository, most of the time, what they want are the releases.

Deciding when to make a new release is at the discretion of the dataset developer. In practice, one makes fewer releases than one does commits into the `git` repository, though there is nothing stopping developers from releasing a new version for every commit. The flexibility here allows developers to do internal work between releases and only release the data to users when the revision represents a clear improvement on the previous release.

Another important consideration is that websites like `GitHub` naturally cater for two types of data users accessing the data: those that interact with the data via point and click downloading, and those that use programmatic interaction (Fig. 1, Table 3). Specifically, `GitHub` releases can be downloaded directly by users or accessed programmatically via the `GitHub` API.

Semantic versioning

To realize the full benefits of a versioned controlled dataset, users should be able to easily intuit the types of changes that have occurred among versions. Since software development has effectively already dealt with a similar problem in the labelling of software releases, we suggest there is benefit in adopting the best-practices from that field.

Specifically, we suggest adapting the process of semantic versioning, developed for labelling successive releases of software (see semver.org), to labelling of successive releases of an evolving dataset (Fig 2). In semantic versioning of software, a tri-digit label of the form "X.Y.Z" is applied to each version,

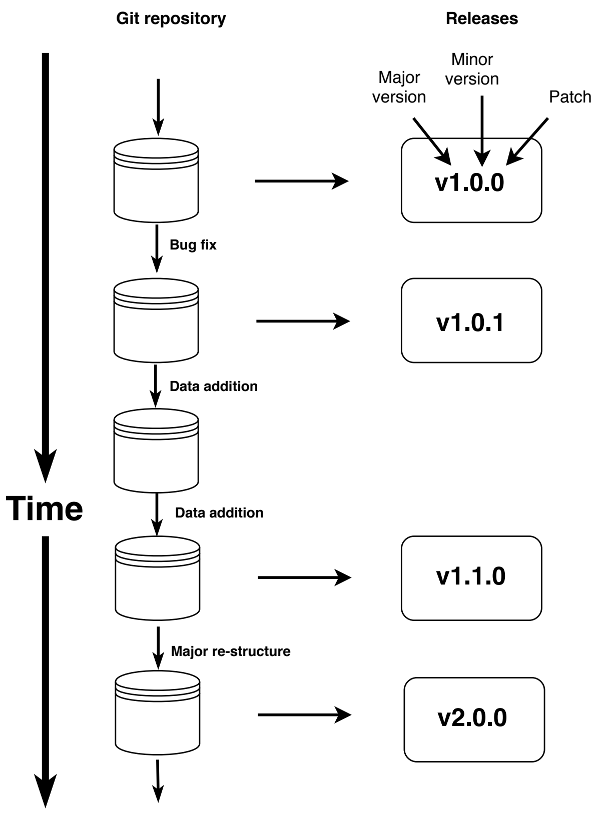

Figure 2. Semantic versioning allows dataset developers to communicate to users the types of changes that have occurred between successive versions of an evolving dataset, using a tri-digit label where increments in a number indicate MAJOR-, MINOR- and PATCH-level changes, respectively. See text for further details.

where X, Y, and Z are non-negative integers. For example, version "2.1.2". Although everyday practice may differ, the guidelines at semver.org suggest labels are incremented in a particular way, determined by changes in the public API for the software.

Though the analogy to software is not perfect, datasets can also be thought of as having an "Interface", determined by the structure of the dataset, which dictates how users interact with the resource. For example, in tabular data the structure is determined by the names of different files, the column labels within each, and the presence of different subgroups within the table (as indicated by labels in particular columns). Successive versions of a dataset can then be labelled in a manner analogous to that of software, determined by the structure of the dataset and changes in that structure.

There are two natural advantages of adapting the process of semantic versioning for dataset development. The first is that enables natural ordering of releases. The second, is that it

Table 2. Example datasets currently delivered using the `datastorr` package for `R`

`GitHub` repository	Dataset description
traitecoevo/taxonlookup	Taxonomy of world's land plants [15].
traitecoevo/growthform	Growth form of world's land plants [21].
traitecoevo/baad.data	Size dimensions of plants for many species from across the world [14].
ecohealthalliance/cites	Trade details from Convention on International Trade in Endangered Species (CITES)
madams1/nbadata	Statistics from the National Basketball Association (NBA) seasons 1996-97 to 2016-17
madams1/floridainmates	Statistics on Florida state's inmate population, from Florida Department of Corrections
traitecoevo/fungaltraits	Traits of world's fungi species [22].

enables developers to signal the type and magnitude of change that occurred in the product between successive versions. Seeing a series of version numbers, users of an evolving dataset know the developer's view on the type and/or magnitude of change between versions.

Drawing inspiration from the guidelines for semantic versioning of software at semver.org, we suggest the following guidelines for labelling of a dataset with semantic versioning:

- Clearly communicate the structure of the dataset in the metadata or landing page. This includes file types, data type, and element names.
- Use versions beginning with "0.Y.Z" to indicate products where the structure is still in development.
- Version "1.0.0" defines the structure.
- Once defined, increment version numbers to communicate any changes to the structure.
- Increment the "Major" version when you make changes to the structure that are likely incompatible with any code written to work with previous versions. Such changes may include revising the file names, the structure of the dataset, or changing element names (e.g. column headers). Substantial additions of data might also be considered a major change to structure, especially where they add new subgroups to the dataset.
- Increment the "Minor" version to communicate any changes to the structure that are likely to be compatible with any code written to work with the previous versions (i.e. allows code to run without error). Such changes might involve adding new data within the existing structure, so that the previous dataset version exists as a subset of the new version. For tabular data, this includes adding columns or rows. On the other hand, removing data should constitute a major version, as records previously relied on may no longer exists.
- Increment the "Patch" version to communicate correction of errors in the actual data, without any changes to the structure. Such changes are unlikely to break, or change analyses written with the previous version in a substantial way.
- Once a dataset version has been released, do not modify it. Further modifications are released under a new version number.

While it is hoped the guidelines above help users in understanding the types of changes that have occurred between successive versions of a dataset, *any* change in a dataset may alter the results of a users' analysis in non-trivial ways. Unlike developers of software, developers of a dataset cannot guarantee full backwards-compatibility, i.e. that certain results will remain unchanged in updated versions. We suggest responsibility for verifying how different versions of an evolving dataset influence their particular analysis or use thus always remains with the user, even if simply applying a so-called "patch". While further work – and likely experience – is needed to refine the process of semantic versioning for datasets to further develop understanding between data developers and data users of what different changes imply, semantic versioning still provides a more nuanced way to communicate from the developer to the user on the types of change they could expect.

Loading data versions directly into `R` using the `datastorr` package

For efficient usage and to aid reproducibility, many users will want access to all versions of any particular dataset programmatically (Table 3). Code to access a stream of `GitHub` releases could be written individually by each user, but this creates an unnecessary technological hurdle. To make it easier for users to access versioned data via code, we developed a new package for the `R` platform, as one of the most prominent platforms for data science [18].

Our package, called `datastorr` (github.com/ropenscilabs/datastorr), facilitates access to releases of any evolving dataset hosted on `GitHub` (Fig. 1). Specifically, the `datastorr` package: 1) Contains the main code needed to interact with the `GitHub` API to retrieve versions of the dataset; and 2) Enables users to construct the shell of a second, dataset-specific `R` package, which can be distributed and used to access releases for a specific repository stored on `GitHub`. Using `datastorr`, a researcher can create and distribute a custom `R` package that facilitates access to their data with (very) minimal computational skills.

For example, `datastorr` has been used to build several packages (Table 2), including `taxonlookup` (github.com/traitecoevo/taxonlookup), which hosts data on the Taxonomy of world's land plants [15]. The R package `taxonlookup` consists of only a few simple functions and associated help files, that were automatically generated with `datastorr`. For a user, accessing a version of the data is a simple as typing a single line of code (Fig. 1). Accessing a different version of the data involves changing only the version number. From the user's perspective, the existence of the `taxonlookup` and `datastorr` packages makes reproducing analyses using specific versions of the data [e.g.][][15, 23] possible.

Using `datastorr`, dataset developers can set up their own `R` package to deliver versions of an evolving dataset simply by providing:

i. a `GitHub` repository name (e.g., "traitecoevo/taxonlookup") where releases are stored;
ii. the filename in the release that contains data;
iii. the function used to load the data file into `R`.

Then as the dataset grows over time, the developers update the `git` repository and create a `GitHub` release with a new version number. All the releases are simultaneously available to any user, both point-and-click and programmatically.

The dataset-specific packages created by `datastorr` are designed to be computationally efficient and also work offline. Packages created by `datastorr` contain no actual data, only the rules for fetching the data. As such, the basic package structure is quick to install and takes up virtually no space on the user's hard-drive. The package functions by fetching each data version once (the first time it is requested), and then caching these files locally for future reuse. Moreover, users can store several versions of an evolving dataset on their computer and unambiguously access different versions with single function.

Discussion

The key issue we are dealing with in this article may be familiar to many readers: many datasets are constantly evolving and, despite tremendous advances in data sharing and associated technologies over the last decade, there is as yet little consensus about how to maintain and distribute multiple versions of an evolving dataset, especially for small research teams. While such teams could in principle create their own dynamic web interface, the technological hurdles, cost and maintenance required are discouraging. Moreover, existing platforms for distributing data offer a limited set of features for the delivery of successive versions of an evolving dataset. This suggests there is a need for an easy, cheap, and scalable solution for maintain-

Table 3. Goals and requirements of different parties involved in creating and using and evolving dataset.

Group	Primary goal	Requirements
Developer	Create and distribute versions of an evolving dataset	Low technical overhead
		Low initial and ongoing cost and maintenance
		Easy workflow for releasing new versions
		Enable user feedback in error checking and contributions
		Long term preservation
Contributor	Contribute to future versions of an evolving dataset	Add new data
		Report errors in existing data
Users (all)	Easy access to all versions of an evolving dataset	Access metadata and background information
		Access to all versions of a dataset
		Ability to give feedback and contribute
		Long term stability
Users (programmatic)	As above, plus	
		Programmatic access to all versions of an evolving dataset
		Reproduce products using specific versions of an evolving dataset
		Easy installation

ing and distributing successive versions of an evolving dataset. By adopting open-source and scalable practices from software development, we believe a workable system already largely exists. To aid this process, we created the `datastorr` package to deliver dataset versions directly into the R environment. The approach and package is already being used to deliver versions of several evolving datasets spanning a wide range of topics (Table 2). Moreover, as it builds off established and open source software and data science platforms (Table 1), the proposed system is already easy to deploy on relatively large scale.

Towards an ecosystem for evolving data

Our contribution here connects with a growing number of recommendations and technologies supporting the sharing and reuse of evolving data. Such contributions include community guidance on good practice in data curation [6, 8], data citation [7] and the FAIR principles for making datasets Findable, Accessible, Interoperable, and Reusable by both machines and humans [11]. In our proposed system, information about appropriate attribution for any dataset (whatever that is determined to be) should be made readily available, either on the landing page within GitHub, or even better distributed as part of the versioned dataset itself. Similarly, datasets can be structured to make them follow the FAIR principles, to the extent possible. Notably, our workflow with the `datastorr` package demands machine access to datasets – a core focus for the FAIR principles. While our proposed workflow does not currently enhance discoverability of new datasets, this is a broad challenge faced by all data platforms and researchers.

While our package `datastorr` offers an easy way for users of the R ecosystem to directly access dataset version, users of other languages can also access the datasets. Moreover, packages similar to `datastorr` would ideally be developed to make accessing dataset versions as easy as it is with `datastorr`.

Within the R ecosystem, the `datastorr` package complements other approaches for creating and delivering datasets. One common approach used within R is to embed data directly within an R package, which can then be distributed via the Comprehensive R Archive Network (cran.r-project.org). Moreover, dedicated packages are being developed to assist dataset developers in creating data packages [24]. An advantage of this approach, compared to ours, is that the data are immediately available in the package (whereas our packages only contain instructions for fetching the data). This advantage however also brings limitations. Notably, datasets must be under 5MB, and only one version of a dataset package can be installed on any given machine at any one time. `datastorr` offers a viable approach for overcoming these limitations.

There are also many emerging or alternative technologies that offer other possible ways to implement a system for storing and distributing versions of an evolving dataset. Our solution currently emphasises the platform GitHub, but similar functions could be achieved via other `git` hosts such as bitbucket.org and gitlab.com. Git repositories can also be extended to accommodate larger files using features like Git-Large File Storage or git-annex. More fundamentally, there are emerging alternatives for version control specifically designed for data, such as the datproject.org, and other new platforms for distributing data, such as the Comprehensive Knowledge Archive Network, (CKAN) and Open Knowledge International, (OKFN).

The key here is not the specific technology, but rather the concept of creating, maintaining, and distributing versions of an evolving dataset, which may be achieved with all of these approaches. Indeed, as with every technology the best available approach is certain to evolve, especially as emerging technologies facilitate even better delivery of data in the future.

Further advantages and extensions

A central feature of the proposed system is that data are maintained on the web. This has two main benefits: first, it provides a platform for multiple data contributors to sync their files and correspond about changes in the dataset, and second, it allows for hosting of a stream of data releases for distribution (Fig. 1). Web platforms thus act as a central point for the collection, curation, and distribution of the data. Additionally, one of the greatest benefits of using web platforms like `GitHub` for development of both software and data has been the way they encourage contributions from multiple individuals working simultaneously — including from people from outside the initial group of project participants [25, 9]. Multiple developers can make changes to different parts of the code (or, in our case, data) and the `git` system will integrate these together or, when needed, flag where there are conflicts that need to be resolved. The proposed system of data delivery thus has the added benefit of facilitating seamless and transparent collaboration among research groups in the construction and maintenance of datasets.

An important concern for any data delivery system is the stability and reliability of the system. In the short term, users

want minimal downtime, high speed, and seamless operation. As one of the largest companies hosting computer source code, `GitHub` provides exceptional performance in this regard – certainly as good or better than nearly any system scientists might build themselves. Thus, short-term concerns of reliable and fast performance are almost guaranteed.

In the long term, scientists want their datasets, software, and papers to preserved and remain accessible. While our proposed system for data delivery does not guarantee long-term preservation, users can also choose to automatically archive data-versions released on `GitHub` version in one of several traditional data archives, with a DOI (Digital Object Identifier) minted for each release. Currently, both `Zenodo` and `FigShare` each integrate with `GitHub` for archiving of material hosted there. Ideally, tools like `datastorr` would also be developed to pull versions from these archives too.

Availability of Supporting Data

Snapshots of the code and example file are archived in the GigaScience GigaDB repository [26].

- Project name: `datastorr`
- Project home page: github.com/ropenscilabs/datastorr.
- Operating system(s): Platform independent
- Programming language: R
- License: MIT

- Project name: `datastorr example`
- Project home page: github.com/richfitz/datastorr.example.
- Operating system(s): Platform independent
- Programming language: R
- License: MIT

- Project name: `taxonlookup`
- Project home page: github.com/traitecoevo/taxonlookup.
- Operating system(s): Platform independent
- Programming language: R
- License: MIT

Declarations

List of abbreviations

API = An Application Programming Interface provides a set of protocols for exchanging information. CKAN = Comprehensive Knowledge Archive Network. DOI = Digital Object Identifier. FAIR = Findable, Accessible, Interoperable, Reusable. Gb = Gigabyte. Mb = Megabyte. OKFN = Open Knowledge International.

Ethical Approval

Not applicable.

Consent for publication

Not applicable.

Competing Interests

The author(s) declare that they have no competing interests.

Funding

DSF was funded by the Australian Research Council (FT160100113). MWP was funded by a NSERC Discovery Grant (RGPIN-2017-04590).

Author's Contributions

RF developed the datastorr package. All authors discussed the concepts and wrote the paper.

Acknowledgements

We thank D Noble for comments on an earlier draft and C Boettiger for helpful discussions.

References

1. Whitlock MC. Data archiving in ecology and evolution: best practices. Trends in Ecology & Evolution 2011;26:61–65.
2. Fairbairn DJ. The advent of mandatory data archiving. Evolution 2011;65:1–2.
3. Piwowar HA, Vision TJ, Whitlock MC. Data archiving is a good investment. Nature 2011;473:285–285.
4. Van Noorden R. Data-sharing: everything on display. Nature 2013;500:243–245.
5. Gibney E, Van Noorden R, Scientists losing data at a rapid rate; 2013. Nature news. doi: 10.1038/nature.2013.14416.
6. Goodman A, Pepe A, Blocker AW, Borgman CL, Cranmer K, Crosas M, et al. Ten simple rules for the care and feeding of scientific data. PLoS Comput Biol 2014;10:e1003542.
7. Data Citation Synthesis Group. Joint declaration of data citation principles. FORCE11 2014;`https://doi.org/10.25490/a97f-egyk`.
8. Lowndes JSS, Best BD, Scarborough C, Afflerbach JC, Frazier MR, O'Hara CC, et al. Our path to better science in less time using open data science tools. Nature Ecology & Evolution 2017;1:0160.
9. Perkel J. Democratic databases: science on GitHub. Nature 2016;538:127–128.
10. Kratz JE, Strasser C. Making data count. Scientific Data 2015;2:sdata201539.
11. Wilkinson MD, Dumontier M, Aalbersberg IJ, Appleton G, Axton M, Baak A, et al. The FAIR guiding principles for scientific data management and stewardship. Scientific Data 2016;3:sdata201618.
12. Yenni GM, Christensen EM, Bledsoe EK, Supp SR, Diaz RM, White EP, et al. Developing a Modern Data Workflow for Regularly Updated Data. PLOS Biology 2019;17:e3000125.
13. Ernest SM, Yenni GM, Allington G, Bledsoe E, Christensen E, Diaz R, et al. The Portal Project: a long-term study of a Chihuahuan desert ecosystem. bioRxiv 2018;p. 332783.
14. Falster DS, Duursma RA, Ishihara MI, Barneche DR, FitzJohn RG, Vårhammar A, et al. BAAD: a Biomass And Allometry Database for woody plants. Ecology 2015;96:1445.
15. Pennell MW, FitzJohn RG, Cornwell WK. A simple approach for maximizing the overlap of phylogenetic and comparative data. Methods in Ecology and Evolution 2015;7:751–758.
16. Abolfathi B, Aguado DS, Aguilar G, Prieto CA, Almeida A, Ananna TT, et al. The fourteenth data release of the Sloan Digital Sky Survey: first spectroscopic data from the extended Baryon oscillation spectroscopic survey and from the second phase of the Apache point observatory galactic

evolution experiment. The Astrophysical Journal Supplement Series 2018;235(2):42.

17. Nielsen LH, Zenodo now supports DOI versioning!; 2017. OpenAIRE blog. https://blogs.openaire.eu/?p=2010.
18. R Core Team. R: a language and environment for statistical computing. R Foundation for Statistical Computing, Vienna, Austria; 2017, http://www.R-project.org/.
19. Perez-Riverol Y, Gatto L, Wang R, Sachsenberg T, Uszkoreit J, da Veiga Leprevost F, et al. Ten simple rules for taking advantage of git and gitHub. PLOS Comput Biol 2016;12:e1004947.
20. Ram K. Git Can Facilitate Greater Reproducibility and Increased Transparency in Science. Source Code for Biology and Medicine 2013;8:7.
21. Taseski G, Beloe CJ, Gallagher RV, Chan JY, Dalrymple RL, Cornwell WK. A Global Growth Form Database for 143,616 Vascular Plant Species. Ecology 2019;in press:e02614.
22. Cornwell WK, Flores-Moreno H, traitecoevo/fungaltraits v0.0.3; 2018. https://doi.org/10.5281/zenodo.1216257.
23. Mounce R, Rivers M, Sharrock S, Smith P, Brockington S. Comparing and contrasting threat assessments of plant species at the global and sub-global level. Biodiversity and Conservation 2018 Mar;27(4):907–930. https://doi.org/10.1007/s10531-017-1472-z.
24. Finak G, Mayer B, Fulp W, Obrecht P, Sato A, Chung E, et al. DataPackageR: Reproducible data preprocessing, standardization and sharing using R/Bioconductor for collaborative data analysis. Gates Open Research 2018;2:31.
25. Rogers M, The Github revolution: why we're all in open source now; 2013. https://www.wired.com/2013/03/github/.
26. Falster DS, Pennell MW, FitzJohn RG, William CK. Supporting Data for "Datastorr: A Workflow and Package for Delivering Successive Versions of 'evolving data' directly into R". GigaScience Database 2019;http://dx.doi.org/10.5524/100573.

Figure 2

Click here to access/download;Figure;Figure

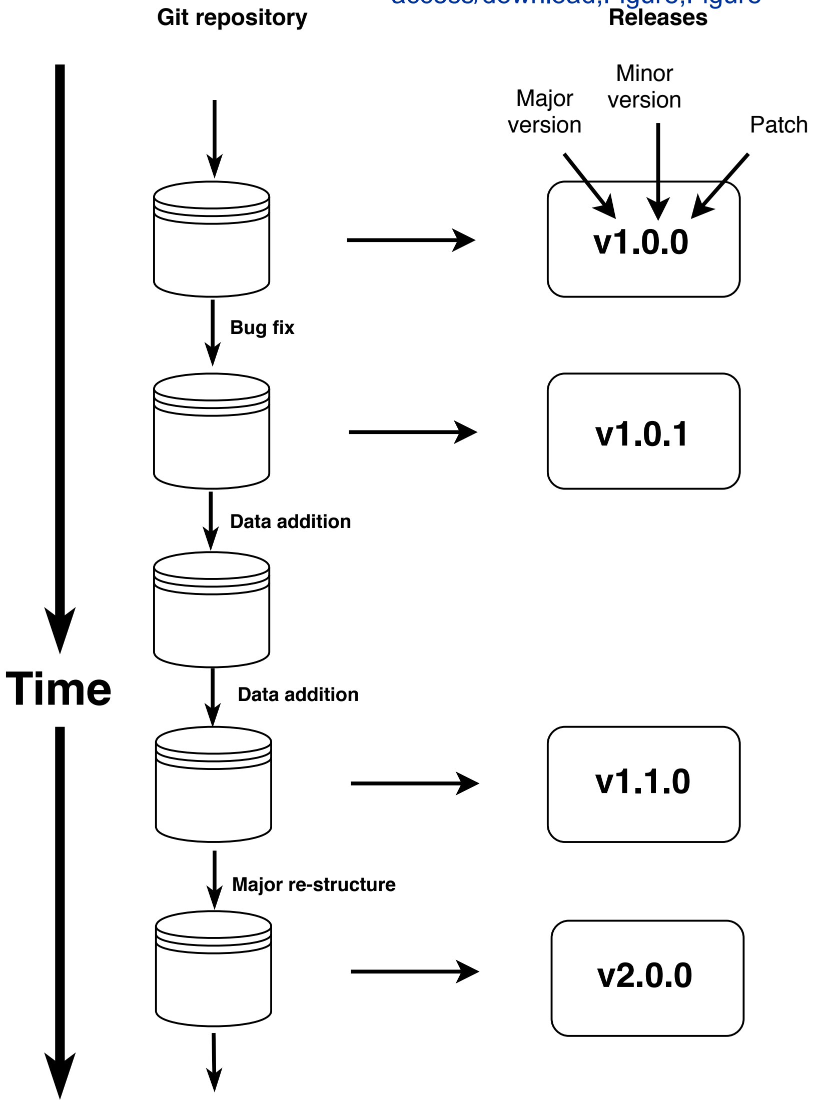

Figure 1

Click here to access/download;Figure;Figure-stack.pdf

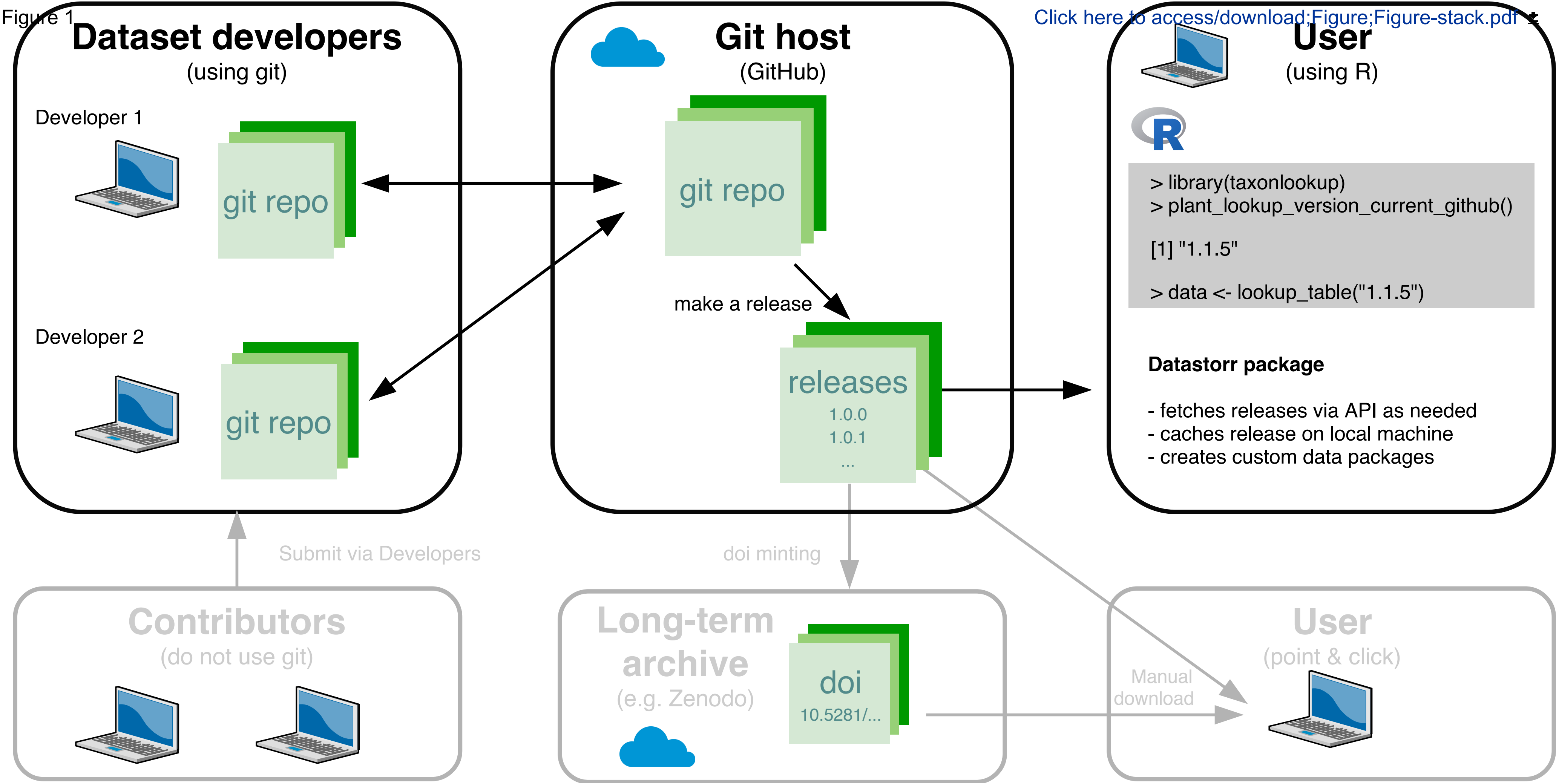

Supplement: GIGA-D-18-00005_Revision_3.pdf [file giz035_giga-d-18-00005_revision_3.pdf]
