# Supplementary material for: Datastorr: a workflow and package for delivering successive versions of 'evolving data' directly into R
Source: Gigascience. 2019 May 1;8(5):giz035. doi: 10.1093/gigascience/giz035 (PMC6506717; doi:10.1093/gigascience/giz035)
Supplement: Response_to_Reviewer_Comments_Original_Submission.pdf [file giz035_response_to_reviewer_comments_original_submission.pdf]

Author's Response To Reviewer Comments

Close

Comments from Reviewer #1:

I think the topic of this paper is important, and the work done here is valuable. However, the paper as written is a bit muddled currently, and is not really well set in the context of related work.

> Thank you for the constructive review. All reviewers commented that the paper should be more focussed, and also needed stronger links to related work. We have followed these suggestions.

The specific foci of the paper and some comments on them are:

1. datasets often need version - this is fine

> Agreed.

2. version numbers - the general idea is fine, but the information about DOIs is a bit unclear. Figure 1 shows a concept DOI. How is this generated? How does it know about the individual version DOIs? Is this using RelationType metadata? Or adding information within the DOI name itself? And how will these DOIs be used? Would a person using a version of a data set cite both the version's DOI and the concept's DOI? The paper does not contain enough discussion about DOIs and their usage.

> Minting of DOI's is an optional extra in our workflow and does not impact on the functioning of our core workflow. We tried to make this clearer in the revised text, by
Highlighting "minting DOI" as an optional in the figure showing the overall workflow.
Removing DOIs from figure about semantic versioning altogether
Noting in the Discussion, that "Minting of DOIs" is an option extension.

3. the VDD system. It's also unclear if this paper is trying to make the case that this is needed, and that it can be provided by multiple packages, or if this is something that is just supported by the authors VDD system. In other words, is this is a general need and a general solution, or is this a specific solution using a specific tool? It seems to be doing a little of both, and it would be better to discuss the general problem first, then a general solution, then the specifics of the solution in VDD, to make this more clear. The penultimate paragraph of the paper does talk about this a little (and it also includes some related work), but it would be better to move this discussion to earlier in the paper. Related to this, it seems that the software discussed here is specific to R. How would a Python user use these ideas?

> As both reviewers note, versioning of datasets is not entirely new. We have therefore focussed our paper more explicitly on the particular workflow we are promoting, by:
In the introduction, framing versioning of datasets as an emerging approach with increasing attention (as opposed to an entirely novel idea)
Removing the previous Table 1, which offered a broader comparison of dataset publishing which did not fit with the narrower focus
Removing the phrase VDD (Versioned Data Delivery) as we have a specific rather than general tool
Including more discussion of related approaches (see Discussion), including other technologies and relevance for users of other languages (eg Python).

In addition:

This work should also be compared with http://git-annex.branchable.com, which shares many elements of this work. In fact, I would like to see a related work section, which could include table 1, more discussion, and potentially discussion of how other types of material with multiple versions are handled, including software and preprints/papers.

> As noted in the previous comment, we removed the previous Table 1. However, the revised discussion includes a broader discussion of related technologies, including git annex.

There could also be some discussion about fields in which data releases are common, and users are used to dealing with them. For example, the data releases for SDSS: http://www.sdss.org/dr14/ and http://www.sdss.org/collaboration/citing-sdss/

> Thanks for this great reference. We have included the SDSS in both the introduction and discussion.

I'm also a little surprised there is no mention of the FAIR data principles (https://doi.org/10.1038/sdata.2016.18) or the data citation principles (https://www.force11.org/group/joint-declaration-data-citation-principles-final) here.

> Agreed. These links are mentioned in the discussion, in the new section "Towards an ecosystem for evolving data"

Comments from Reviewer #2:

I think this is an interesting approach. I sense that the authors are being slightly disingenuous about the current state of affairs in order to make their solution more attractive, but there is no need of it.

> Thank you for the constructive review. During the process of review we have discovered multiple resources that indicate the general idea of versioning of data is not as new as we portrayed it to be. We have therefore revised the paper to focus more on our particular workflow, which assists with distributing versions of an evolving dataset.

Specific points:
- "The current model for publishing data has not yet embraced the idea that many datasets are designed to answer scientific questions that extend beyond the scope of a single empirical paper. These datasets are constantly evolving." While it may be true that all dynamic datasets are intended as a resource for multiple papers (otherwise why bother), the reverse is not true. There are plenty of static datasets that are provided explicitly as an ongoing resource for future research: major social surveys, outputs of astronomical missions, etc.

> Agreed. This sentence has been revised to read "In particular, in some areas, such as our own area of ecology and evolution, we are only starting to support the fact that some high-quality datasets may be evolving entities". Moreover, in the revised text we have tried to remove comments about general publishing of data and instead focus on instances where dataset developers want to distribute versions.

- "Yet, current models for publishing datasets do not facilitate distributing successive versions of a dataset." A similar point is made in Table 1. This is false. In our institutional data repository, based on Eprints, we can provide successive linked versions of a dataset. Both Zenodo and FigShare, later mentioned as providing the DOI minting and long-term archiving part of the proposed solution, can do likewise. I'm sure other platforms can as well.

> Agreed. The revises text explicitly acknowledges this fact (see Introduction)

I would much prefer it if the authors were clearer about what they are actually proposing here: a simple solution for research groups to maintain a dynamic dataset, and integration of this solution into R. It has been said that there is a difference between lowercase publication (public distribution) and uppercase Publication (guarantees of stability and long-term availability). This paper positions itself as addressing the latter but is in fact addressing the former, and indeed for Publication relies on the very solutions it dismisses in the introduction. As I say, there is no need of this. The solution is interesting enough to stand on its own merits, and I rather like it.

> Thank you. We have taken this suggestion on wholeheartedly and have reorganized the paper accordingly. The title, abstract, key points, introduction and discussion have all been reworked to focus on the key innovation, as identified by the reviewer: a simple solution for research groups to maintain a dynamic dataset, and integration of this solution into R

On a technical point, certain tricks can be used with Git to get meaningful diffs with some types of

binary files (e.g. zipped XML file formats), but this is probably beyond the detail needed in this paper. The point about binary files is that using Git with them (in the absence of such tricks) does not give you any advantage over the regular linked snapshot approach used by data repository platforms.

> We added a minor note to this effect in the revised text (see section “version control”).

Comments from Reviewer #3:
This paper describes a problem of importance and contemporary interest (versioning scientific datasets) and presents a practical and effective approach. Generally I like the authors' solution and presentation of the problem, and I think the paper makes a useful contribution.

> Thank you for the constructive review.

The introduction (top of p. 2) is perhaps a bit overstated. Figshare (https://knowledge.figshare.com/articles/item/can-i-edit-or-delete-my-research-after-it-has-been-made-public), Dryad (https://datadryad.org//pages/policies#versioning), and Zenodo (http://help.zenodo.org/#versioning) do support versioning. Their approaches to versioning are perhaps not the most flexible (Dryad's is particularly limited of the three), but nevertheless it's not correct to say that datasets stored in those systems are entirely static. Perhaps the authors could revise the statement.

> Agreed. This text has been revised, as noted above in response to similar comments from R1 and R2.

My one criticism is that I don't think it is obvious how to translate semantic versioning from software to data. In the case of software, versioning benefits from an implicit sense of correctness. I say only a "sense," because the semantics of a piece of software are never formally specified except in very rare circumstances. Nevertheless, there is almost always an implied sense that the software is supposed to perform certain functions and give certain results, and people use the software and develop against the software making those implicit assumptions. This enables the common understanding that the fix of a bug in the software can usually be considered a patch change (1.0.0 -> 1.0.1) because the software's users were already expecting the software to behave that way, and so upgrading the software generally can't cause any harm. But in the case of data, what constitutes a patch change? A discovery that, say, the calibration of a sensor was mis-set, and a subsequent correction of that calibration, could be interpreted as a simple bug fix since it affects the "correctness" of the data: the calibration and the data were supposed to have the new values all along. And yet, the existence of that bug and its correction may be of huge importance: it may invalidate all previous uses of the data! The authors of this paper describe a patch change as "a small error correction which is unlikely to break, or change in a substantial way, a users' analyses". But which changes will break downstream analyses, and which will not? Developing an understanding between data producers and data consumers of what semantic versioning means for data, and how it is routinely applied, is perhaps outside the scope of this paper, but I think at minimum the authors should acknowledge this as an area of needed future work.

->This is a fantastic comment, which caused us to discuss our application of semantic versioning in more and revise our recommendations. The reviewer is right that the mapping from software to dataset is not perfect. In software the version numbers refer to changes in the public interface for the software (i.e. it’s API). In our revised text we apply this idea of an Interface to the dataset and recommend version increments according to how they change this interface. We agree with the reviewer is absolutely right that dataset developers cannot guarantee an apparently “Minor” or “Patch increment won’t have non-trivial impacts on prior work. We now acknowledge and discuss this.

As a general rule, I would request that acronyms (e.g., "CKAN, OKFN or Git-LFS") be hyperlinked to their definitions. Otherwise it's just alphabet soup.

> Agreed, done.

Typos/grammatical errors:

p. 1 line 53: dataset on a biological organisms
p. 2 line 44: by adding multiple version of
p. 2 line 9: on a users local computer
p. 2 line 37: In it's present
p. 3 line 61: a users' analyses

p. 4 line 10: From the users perspective
p. 4 line 14: to deliver there versioned dataset

> Fixed, thank you!

Close
