# Supplementary material for: Datastorr: a workflow and package for delivering successive versions of 'evolving data' directly into R
Source: Gigascience. 2019 May 1;8(5):giz035. doi: 10.1093/gigascience/giz035 (PMC6506717; doi:10.1093/gigascience/giz035)
Supplement: Response_to_Reviewer_Comments_Revision_1.pdf [file giz035_response_to_reviewer_comments_revision_1.pdf]

Author's Response To Reviewer Comments

Close

Comments from Reviewer #1:

This version of the paper is much better than the first one, but still needs more work.

> Thank you for the constructive review. We have followed the reviewer's suggestions and outline our responses in detail below.

As one of the other reviewers stated in a previous review, there is a difference between publishing datasets (archiving, etc.) and sharing datasets (distributing, etc.). The introduction still confuses these. For example, the SDSS work is publishing, but the solution here is limited to sharing.

> Thank you. We have edited the introduction further to emphasise that our solution deals with distribution/sharing and not publication of data. As you may be aware, previous reviewers asked us to acknowledge the SDSS as a leading example where versioning has been addressed, which we have done. We have therefore kept this text, but with changes as noted above.

GitHub was not created for storing data, though limited data storage certainly happens there. I could imaging that if this work became more popular, GitHub might try to prevent it from working. This makes me wonder about what this project would do if GitHub was not available to be used in this way.

> The ability to host "Releases" is a core feature of Github. While it is possible they will stop supporting our use case, it is hard to imagine this occurring. If it did occur, there are several options, such as : shifting to use the "Downloads" feature offered through Bitbucket, or storing the built material in the repository itself and storing this on either Github or Bitbucket.

The recommendation for "Saving all files as plain text, so that git can identify line- by-line changes. For example, save tabular data as a 'csv'" is problematic in many cases, as it leads to increased file size, increased time to move the files, and increased time for operations.

> As data publishers ourselves, we personally have found the plain-text approach VERY useful, as it allows us to use git's version control functionality to identify changes. But the reviewer is right, it could cause problems with large files. We have therefore amended the text to make it clear that that storing files as text is not essential and may become problematic for very large data files.The reviewer will be relieved to know that releases are stored in zip format, so the extra costs they note are avoided.

The comma in "Of particular interest for current purposes, is" should be removed.

> Done.

The comment about semantic versioning and software API changes simply is not correct. While API changes most often do occur in changes of the major version, many major versions do not include any API changes, and API changes may also occur in minor versions (though not very often).

> We appreciate the reviewer feels our description of the system used to label software versions relative to changes in the API is incorrect. However, our statements on this topic reflect the guidelines given in the cited reference for semantic versioning (http://semver.org/). For example, on the first line of that site it is stated "MAJOR version when you make incompatible API changes". And then further down, "The way in which the version number is incremented after this release is dependent on this public API and how it changes" (see here). We are therefore not convinced our text is incorrect. But we can readily agree not all developers follow this system exactly. Hence we have reworded the text in this section to make it clear that this is just one suggested option.

The idea of an Interface for a dataset is not really clear, and is probably not well-named. Maybe

"Structure" would be better?

> Agree. In the revised text, we only use the term “Interface” once and in quotes, and thereafter refer to the “structure” of the dataset. We feel it helpful to keep this single usage of the word “Interface” to connect with the discussion about software, where the term Interface is used, as the last word in the term API.

an in "Interface an then be labelled " should be can.

> Done

The definition of semantic versioning for software does not label X, Y, and Z, but the later discussion about using it in data does name it, without tying these names back to the labels or positions.

> We’re sorry, but we didn’t understand exactly what the reviewer was requesting with this comment.

I also disagree with the idea that a change in the "Interface" is the reason for a change in the major version. As with software, it's very possible that a major change in the data values could be considered a major version.

> We amended the text to make clear that “Substantial additions of data might also be considered a major change”.

So little of this paper actually is about R that perhaps the title should be changed.

> We appreciate the Reviewer's suggestion but have not followed their suggestion, as it conflicts with suggestions made in the previous round of review. As the editor may recall, the version initially submitted had a broader title, but the reviewer's requested we narrow the focus to what we were “actually proposing: a simple solution for research groups to maintain a dynamic dataset, and integration of this solution into R” .

"A central feature of the proposed system is that data are maintained in the cloud" seems incorrect to me. I think saying "on the Internet" or "on the web" would be more accurate.

> Done. Reworded to say “data are maintained on the web”. Similarly changes were implemented throughout the paragraph.

Close
