# Supplementary material for: Datastorr: a workflow and package for delivering successive versions of 'evolving data' directly into R
Source: Gigascience. 2019 May 1;8(5):giz035. doi: 10.1093/gigascience/giz035 (PMC6506717; doi:10.1093/gigascience/giz035)
Supplement: Response_to_Reviewer_Comments_Revision_2.pdf [file giz035_response_to_reviewer_comments_revision_2.pdf]

Author's Response To Reviewer Comments

Close

Minor changes as requested, including

- add phone for correpsonding author
- add ORCID ids
- add link to supporting data package
- add details for two other supporting software packages included in supporting data package
- add extra items to List of abbreviations
- add grant numbers
- references: add URLS and update paper now published. Reference data package

Close
