# Supplementary material for: Datastorr: a workflow and package for delivering successive versions of 'evolving data' directly into R
Source: Gigascience. 2019 May 1;8(5):giz035. doi: 10.1093/gigascience/giz035 (PMC6506717; doi:10.1093/gigascience/giz035)
Supplement: Reviewer_1_Report_Original_Submission -- Daniel Katz — 1/14/2018 Reviewed [file giz035_reviewer_1_report_original_submission.pdf]

Reviewer Report

Title: Datastorr: a workflow and package for delivering successive versions of 'evolving data' directly into R

Version: Original Submission **Date:** 1/14/2018

Reviewer name: Daniel Katz

Reviewer Comments to Author:

I think the topic of this paper is important, and the work done here is valuable. However, the paper as written is a bit muddled currently, and is not really well set in the context of related work.
The specific foci of the paper and some comments on them are:
1. datasets often need version - this is fine
2. version numbers - the general idea is fine, but the information about DOIs is a bit unclear. Figure 1 shows a concept DOI. How is this generated? How does it know about the individual version DOIs? Is this using RelationType metadata? Or adding information within the DOI name itself? And how will these DOIs be used? Would a person using a version of a data set cite both the version's DOI and the concept's DOI? The paper does not contain enough discussion about DOIs and their usage.
3. the VDD system. It's also unclear if this paper is trying to make the case that this is needed, and that it can be provided by multiple packages, or if this is something that is just supported by the authors VDD system. In other words, is this is a general need and a general solution, or is this a specific solution using a specific tool? It seems to be doing a little of both, and it would be better to discuss the general problem first, then a general solution, then the specifics of the solution in VDD, to make this more clear. The penultimate paragraph of the paper does talk about this a little (and it also includes some related work), but it would be better to move this discussion to earlier in the paper. Related to this, it seems that the software discussed here is specific to R. How would a Python user use these ideas?
In addition:
This work should also be compared with http://git-annex.branchable.com, which shares many elements of this work. In fact, I would like to see a related work section, which could include table 1, more discussion, and potentially discussion of how other types of material with multiple versions are handled, including software and preprints/papers.
There could also be some discussion about fields in which data releases are common, and users are used to dealing with them. For example, the data releases for SDSS: http://www.sdss.org/dr14/ and http://www.sdss.org/collaboration/citing-sdss/
I'm also a little surprised there is no mention of the FAIR data principles (https://doi.org/10.1038/sdata.2016.18) or the data citation principles (https://www.force11.org/group/joint-declaration-data-citation-principles-final) here.

Level of Interest

Please indicate how interesting you found the manuscript: Choose an item.

Quality of Written English

Please indicate the quality of language in the manuscript: Acceptable

Declaration of Competing Interests

Please complete a declaration of competing interests, considering the following questions:

- Have you in the past five years received reimbursements, fees, funding, or salary from an organisation that may in any way gain or lose financially from the publication of this manuscript, either now or in the future?
- Do you hold any stocks or shares in an organisation that may in any way gain or lose financially from the publication of this manuscript, either now or in the future?
- Do you hold or are you currently applying for any patents relating to the content of the manuscript?
- Have you received reimbursements, fees, funding, or salary from an organization that holds or has applied for patents relating to the content of the manuscript?
- Do you have any other financial competing interests?
- Do you have any non-financial competing interests in relation to this paper?

If you can answer no to all of the above, write 'I declare that I have no competing interests' below. If your reply is yes to any, please give details below.

I was partially funded by rOpenSci for 1 year to help the project with its financial sustainability, about 1 year ago. This work seems to be associated with rOpenSci (the code is in the ropenscilabs project on GitHub). However I did not know of this work or the authors before I accepted this review.

I agree to the open peer review policy of the journal. I understand that my name will be included on my report to the authors and, if the manuscript is accepted for publication, my named report including any attachments I upload will be posted on the website along with the authors' responses. I agree for my report to be made available under an Open Access Creative Commons CC-BY license (http://creativecommons.org/licenses/by/4.0/). I understand that any comments which I do not wish to be included in my named report can be included as confidential comments to the editors, which will not be published.

I agree to the open peer review policy of the journal

To further support our reviewers, we have joined with Publons, where you can gain additional credit to further highlight your hard work (see: https://publons.com/journal/530/gigascience). On publication of this paper, your review will be automatically added to Publons, you can then choose whether or not to claim your Publons credit. I understand this statement.

Yes Choose an item.
