# Supplementary material for: Datastorr: a workflow and package for delivering successive versions of 'evolving data' directly into R
Source: Gigascience. 2019 May 1;8(5):giz035. doi: 10.1093/gigascience/giz035 (PMC6506717; doi:10.1093/gigascience/giz035)
Supplement: Reviewer_1_Report_Revision_1 -- Daniel Katz — 10/30/2018 Reviewed [file giz035_reviewer_1_report_revision_1.pdf]

Reviewer Report

Title: Datastorr: a workflow and package for delivering successive versions of 'evolving data' directly into R

Version: Revision 1 **Date:** 10/30/2018

Reviewer name: Daniel Katz

Reviewer Comments to Author:

This version of the paper is much better than the first one, but still needs more work.
As one of the other reviewers stated in a previous review, there is a difference between publishing datasets (archiving, etc.) and sharing datasets (distributing, etc.). The introduction still confuses these. For example, the SDSS work is publishing, but the solution here is limited to sharing.
GitHub was not created for storing data, though limited data storage certainly happens there. I could imaging that if this work became more popular, GitHub might try to prevent it from working. This makes me wonder about what this project would do if GitHub was not available to be used in this way.
The recommendation for "Saving all files as plain text, so that git can identify line- by-line changes. For example, save tabular data as a 'csv'" is problematic in many cases, as it leads to increased file size, increased time to move the files, and increased time for operations.
The comma in "Of particular interest for current purposes, is" should be removed.
The comment about semantic versioning and software API changes simply is not correct. While API changes most often do occur in changes of the major version, many major versions do not include any API changes, and API changes may also occur in minor versions (though not very often).
The idea of an Interface for a dataset is not really clear, and is probably not well-named. Maybe "Structure" would be better?
an in "Interface an then be labelled " should be can.
The definition of semantic versioning for software does not label X, Y, and Z, but the later discussion about using it in data does name it, without tying these names back to the labels or positions.
I also disagree with the idea that a change in the "Interface" is the reason for a change in the major version. As with software, it's very possible that a major change in the data values could be considered a major version.
So little of this paper actually is about R that perhaps the title should be changed.
"A central feature of the proposed system is that data are maintained in the cloud" seems incorrect to me. I think saying "on the Internet" or "on the web" would be more accurate.

Level of Interest

Please indicate how interesting you found the manuscript: Choose an item.

Quality of Written English

Please indicate the quality of language in the manuscript: Choose an item.

Declaration of Competing Interests

Please complete a declaration of competing interests, considering the following questions:

- Have you in the past five years received reimbursements, fees, funding, or salary from an organisation that may in any way gain or lose financially from the publication of this manuscript, either now or in the future?
- Do you hold any stocks or shares in an organisation that may in any way gain or lose financially from the publication of this manuscript, either now or in the future?
- Do you hold or are you currently applying for any patents relating to the content of the manuscript?
- Have you received reimbursements, fees, funding, or salary from an organization that holds or has applied for patents relating to the content of the manuscript?
- Do you have any other financial competing interests?
- Do you have any non-financial competing interests in relation to this paper?

If you can answer no to all of the above, write 'I declare that I have no competing interests' below. If your reply is yes to any, please give details below.

I declare that I have no competing interests

I agree to the open peer review policy of the journal. I understand that my name will be included on my report to the authors and, if the manuscript is accepted for publication, my named report including any attachments I upload will be posted on the website along with the authors' responses. I agree for my report to be made available under an Open Access Creative Commons CC-BY license (http://creativecommons.org/licenses/by/4.0/). I understand that any comments which I do not wish to be included in my named report can be included as confidential comments to the editors, which will not be published.

I agree to the open peer review policy of the journal

To further support our reviewers, we have joined with Publons, where you can gain additional credit to further highlight your hard work (see: https://publons.com/journal/530/gigascience). On publication of this paper, your review will be automatically added to Publons, you can then choose whether or not to claim your Publons credit. I understand this statement.

Yes Choose an item.
