# Supplementary material for: Datastorr: a workflow and package for delivering successive versions of 'evolving data' directly into R
Source: Gigascience. 2019 May 1;8(5):giz035. doi: 10.1093/gigascience/giz035 (PMC6506717; doi:10.1093/gigascience/giz035)
Supplement: Reviewer_2_Report_Original_Submission -- Alex Ball — 3/20/2018 Reviewed [file giz035_reviewer_2_report_original_submission.pdf]

Reviewer Report

Title: Datastorr: a workflow and package for delivering successive versions of 'evolving data' directly into R

Version: Original Submission **Date:** 3/20/2018

Reviewer name: Alex Ball

Reviewer Comments to Author:

I think this is an interesting approach. I sense that the authors are being slightly disingenuous about the current state of affairs in order to make their solution more attractive, but there is no need of it. Specific points:
- "The current model for publishing data has not yet embraced the idea that many datasets are designed to answer scientific questions that extend beyond the scope of a single empirical paper. These datasets are constantly evolving." While it may be true that all dynamic datasets are intended as a resource for multiple papers (otherwise why bother), the reverse is not true. There are plenty of static datasets that are provided explicitly as an ongoing resource for future research: major social surveys, outputs of astronomical missions, etc.
- "Yet, current models for publishing datasets do not facilitate distributing successive versions of a dataset." A similar point is made in Table 1. This is false. In our institutional data repository, based on Eprints, we can provide successive linked versions of a dataset. Both Zenodo and FigShare, later mentioned as providing the DOI minting and long-term archiving part of the proposed solution, can do likewise. I'm sure other platforms can as well.
I would much prefer it if the authors were clearer about what they are actually proposing here: a simple solution for research groups to maintain a dynamic dataset, and integration of this solution into R. It has been said that there is a difference between lowercase publication (public distribution) and uppercase Publication (guarantees of stability and long-term availability). This paper positions itself as addressing the latter but is in fact addressing the former, and indeed for Publication relies on the very solutions it dismisses in the introduction. As I say, there is no need of this. The solution is interesting enough to stand on its own merits, and I rather like it.
On a technical point, certain tricks can be used with Git to get meaningful diffs with some types of binary files (e.g. zipped XML file formats), but this is probably beyond the detail needed in this paper. The point about binary files is that using Git with them (in the absence of such tricks) does not give you any advantage over the regular linked snapshot approach used by data repository platforms.

Level of Interest

Please indicate how interesting you found the manuscript: Choose an item.

Quality of Written English

Please indicate the quality of language in the manuscript: Acceptable

Declaration of Competing Interests

Please complete a declaration of competing interests, considering the following questions:

- Have you in the past five years received reimbursements, fees, funding, or salary from an organisation that may in any way gain or lose financially from the publication of this manuscript, either now or in the future?
- Do you hold any stocks or shares in an organisation that may in any way gain or lose financially from the publication of this manuscript, either now or in the future?
- Do you hold or are you currently applying for any patents relating to the content of the manuscript?
- Have you received reimbursements, fees, funding, or salary from an organization that holds or has applied for patents relating to the content of the manuscript?
- Do you have any other financial competing interests?
- Do you have any non-financial competing interests in relation to this paper?

If you can answer no to all of the above, write 'I declare that I have no competing interests' below. If your reply is yes to any, please give details below.

I declare that I have no competing interests

I agree to the open peer review policy of the journal. I understand that my name will be included on my report to the authors and, if the manuscript is accepted for publication, my named report including any attachments I upload will be posted on the website along with the authors' responses. I agree for my report to be made available under an Open Access Creative Commons CC-BY license (http://creativecommons.org/licenses/by/4.0/). I understand that any comments which I do not wish to be included in my named report can be included as confidential comments to the editors, which will not be published.

I agree to the open peer review policy of the journal

To further support our reviewers, we have joined with Publons, where you can gain additional credit to further highlight your hard work (see: https://publons.com/journal/530/gigascience). On publication of this paper, your review will be automatically added to Publons, you can then choose whether or not to claim your Publons credit. I understand this statement.

Yes Choose an item.
