# Supplementary material for: Datastorr: a workflow and package for delivering successive versions of 'evolving data' directly into R
Source: Gigascience. 2019 May 1;8(5):giz035. doi: 10.1093/gigascience/giz035 (PMC6506717; doi:10.1093/gigascience/giz035)
Supplement: Reviewer_3_Report_Original_Submission -- Greg Janée — 3/26/2018 Reviewed [file giz035_reviewer_3_report_original_submission.pdf]

Reviewer Report

Title: Datastorr: a workflow and package for delivering successive versions of 'evolving data' directly into R

Version: Original Submission **Date:** 3/26/2018

Reviewer name: Greg JanÃ©e

Reviewer Comments to Author:

This paper describes a problem of importance and contemporary interest (versioning scientific datasets) and presents a practical and effective approach. Generally I like the authors' solution and presentation of the problem, and I think the paper makes a useful contribution.

The introduction (top of p. 2) is perhaps a bit overstated. Figshare (https://knowledge.figshare.com/articles/item/can-i-edit-or-delete-my-research-after-it-has-been-made-public), Dryad (https://datadryad.org//pages/policies#versioning), and Zenodo (http://help.zenodo.org/#versioning) do support versioning. Their approaches to versioning are perhaps not the most flexible (Dryad's is particularly limited of the three), but nevertheless it's not correct to say that datasets stored in those systems are entirely static. Perhaps the authors could revise the statement.

My one criticism is that I don't think it is obvious how to translate semantic versioning from software to data. In the case of software, versioning benefits from an implicit sense of correctness. I say only a "sense," because the semantics of a piece of software are never formally specified except in very rare circumstances. Nevertheless, there is almost always an implied sense that the software is supposed to perform certain functions and give certain results, and people use the software and develop against the software making those implicit assumptions. This enables the common understanding that the fix of a bug in the software can usually be considered a patch change (1.0.0 -> 1.0.1) because the software's users were already expecting the software to behave that way, and so upgrading the software generally can't cause any harm. But in the case of data, what constitutes a patch change? A discovery that, say, the calibration of a sensor was mis-set, and a subsequent correction of that calibration, could be interpreted as a simple bug fix since it affects the "correctness" of the data: the calibration and the data were supposed to have the new values all along. And yet, the existence of that bug and its correction may be of huge importance: it may invalidate all previous uses of the data! The authors of this paper describe a patch change as "a small error correction which is unlikely to break, or change in a substantial way, a users' analyses". But which changes will break downstream analyses, and which will not? Developing an understanding between data producers and data consumers of what semantic versioning means for data, and how it is routinely applied, is perhaps outside the scope of this paper, but I think at minimum the authors should acknowledge this as an area of needed future work.

As a general rule, I would request that acronyms (e.g., "CKAN, OKFN or Git-LFS") be hyperlinked to their definitions. Otherwise it's just alphabet soup.

Typos/grammatical errors:

p. 1 line 53: dataset on a biological organisms

p. 2 line 44: by adding multiple version of

p. 2 line 9: on a users local computer
p. 2 line 37: In it's present
p. 3 line 61: a users' analyses
p. 4 line 10: From the users perspective
p. 4 line 14: to deliver there versioned dataset

Level of Interest

Please indicate how interesting you found the manuscript: Choose an item.

Quality of Written English

Please indicate the quality of language in the manuscript: Needs some language corrections before being published

Declaration of Competing Interests

Please complete a declaration of competing interests, considering the following questions:

- Have you in the past five years received reimbursements, fees, funding, or salary from an organisation that may in any way gain or lose financially from the publication of this manuscript, either now or in the future?
- Do you hold any stocks or shares in an organisation that may in any way gain or lose financially from the publication of this manuscript, either now or in the future?
- Do you hold or are you currently applying for any patents relating to the content of the manuscript?
- Have you received reimbursements, fees, funding, or salary from an organization that holds or has applied for patents relating to the content of the manuscript?
- Do you have any other financial competing interests?
- Do you have any non-financial competing interests in relation to this paper?

If you can answer no to all of the above, write 'I declare that I have no competing interests' below. If your reply is yes to any, please give details below.

I declare that I have no competing interests

I agree to the open peer review policy of the journal. I understand that my name will be included on my report to the authors and, if the manuscript is accepted for publication, my named report including any attachments I upload will be posted on the website along with the authors' responses. I agree for my report to be made available under an Open Access Creative Commons CC-BY license (http://creativecommons.org/licenses/by/4.0/). I understand that any comments which I do not wish to

be included in my named report can be included as confidential comments to the editors, which will not be published.

I agree to the open peer review policy of the journal

To further support our reviewers, we have joined with Publons, where you can gain additional credit to further highlight your hard work (see: https://publons.com/journal/530/gigascience). On publication of this paper, your review will be automatically added to Publons, you can then choose whether or not to claim your Publons credit. I understand this statement.

Yes Choose an item.
